# Human‐geographic effects on variations in the population genetics of *Sinotaia quadrata* (Gastropoda: Viviparidae) that historically migrated from continental East Asia to Japan

**DOI:** 10.1002/ece3.6456

**Published:** 2020-07-15

**Authors:** Bin Ye, Takumi Saito, Takahiro Hirano, Zhengzhong Dong, Van Tu Do, Satoshi Chiba

**Affiliations:** ^1^ Graduate School of Life Sciences Tohoku University Sendai Japan; ^2^ Department of Biology Faculty of Science Toho University Funabashi Japan; ^3^ Center for Northeast Asian Studies Tohoku University Sendai Japan; ^4^ Agricultural Experiment Station Zhejiang University Hangzhou China; ^5^ Institute of Ecology and Biological Resources Vietnam Academy of Science and Technology Ha Noi Vietnam; ^6^ Graduate University of Science and Technology Vietnam Academy of Science and Technology Ha Noi Vietnam

**Keywords:** biogeography, demographic history, geographic isolation, human effects

## Abstract

**Background:**

Anthropogenic factors potentially affect observed biogeographical patterns in population genetics, but the effects of ancient human activities on the original patterns created by natural processes are unknown. *Sinotaia quadrata*, a widely distributed freshwater snail species in East Asia, was used to investigate this issue. It is unclear whether *S. quadrata* in Japan was introduced from China and how different human uses and varying geographic patterns affect the contemporary population genetics between the two regions. Thus, we investigated the demography of *S. quadrata* and detected its genetic structure in Japan and continental East Asia.

**Results:**

*Sinotaia quadrata* populations first naturally migrated from continental East Asia to Japan, which is associated with the ancient period in Japanese geohistory (about 70,000 years ago). They were then artificially introduced in association with agriculture expansion by human movements in two recent periods (about 8,000 and 1,200 years ago). Populations in different parts of Japan have their own sources. Natural migration in the ancient period and artificial introduction in the recent period suggest that the population distribution is affected by both the geohistory of East Asia and the history of human expansion. In the background of the historical migration and introduction, contemporary populations in the two regions show different genetic patterns. Population divergence levels were significantly correlated with geographical patterns in Japan and significantly correlated with human interventions variables in continental East Asia, suggesting that long‐term geographical isolation is likely the major factor that shaped the contemporary population genetics in Japan, while modern human uses are likely the major factor in continental East Asia.

**Conclusions:**

Our preliminary results show a complex demography and unusual genetic patterns in the contemporary populations for a common freshwater snail and are of significance to determine the historical formation and contemporary patterns of biogeography in Japan and continental East Asia.

## Graphical abstract

We document the population history and genetic history of the common freshwater snail *Sinotaia quadrata* in Japan and continental East Asia. Natural migration in the ancient period and artificial introduction in the recent period suggest that the population distribution is affected by both the geohistory of East Asia and the history of human expansion. Long‐term geographical isolation is likely the major factor that shaped the genetic structure of contemporary populations in Japan, while modern human uses are likely the major factor in continental East Asia.

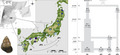

## 1 BACKGROUND

Population genetics analysis is a basic step to understanding genetic variation and evolution processes in recent populations. Comparing studies of population genetics between or among the origin and introduced areas can clarify the migration, gene flow, mutation, and introduction history (Lounnas et al., 2017). It is typically believed that source populations are genetically more structured than introduced populations, and introduced populations might lose genetic diversity because of demographic bottlenecks or founder effects (Dlugosch & Parker, 2008; Dybdahl & Drown, 2011; Mergeay, Verschuren, & De Meester, 2006; Vogel, Pedersen, Giraud, Krieger, & Keller, 2010). However, this would not be the case if there were differences caused by the influence of human activity on the populations between the source and introduced regions, especially for species with low active dispersal ability and for ancient introduction by human activity. Because human‐vectored dispersal enhances gene flow, modifies dispersal paths, and has a variety of effects on genetic exchanges between populations, more human‐vectored dispersal means more genetic mixing, which yields a uniform population structure; this contributes to fitness and persistence of the populations (Bullock et al., 2018; Crispo, Moore, Lee‐Yaw, Gray, & Haller, 2011; Pelletier & Coltman, 2018; Rhymer & Simberloff, 1996). The source populations would be more uniform if humans in the source regions frequently used and transported the focal species. Conversely, populations are more likely to be structured if humans rarely disturb the populations in the introduced regions, and the difference in structure would additionally be facilitated if the geographical patterns were more varied. Therefore, it is worth investigating how human activity, which is sometimes driven by different cultures in different regions, affects population genetic structures by natural environments and geographical patterns.

Organisms in both Japan and continental East Asia, in particular China, are an excellent model system to address these issues, because many connections and differences coexist in the two regions. Particularly, *Sinotaia quadrata* (Benson, 1842) is a widespread freshwater snail species that is distributed in East Asia (Hirano, Saito, & Chiba, 2015; Hirano, Saito, Tsunamoto, Koseki, Prozorova, et al., 2019; Shu, Wang, Cui, & Wang, 2014). It is commonly referred to by the synonym *Bellamya quadrata* (Benson, 1842; Liu, Zhang, Wang, Wang, & Ma, 1979; Qian, Fang, & He, 2014), and it is potentially the same species as *Bellamya aeruginosa* (Reeve, 1863) and *Bellamya purificata* (Heude, 1890) (Cianfanelli et al., 2017; Park, 2015; Wu, Ouyang, Yl, Wang, & Yu, 2000). Although these synonyms were still used in some publications (e.g. Gu, Husemann, Ding, Luo, & Xiong, 2015a; Gu, Zhang, et al., 2015; Gu, Zhou, et al., 2015), it is equally deserving of reassessment for erroneous placements of the Asian viviparids in genus *Bellamya* that is restricted in Africa (Sengupta, Kristensen, Madsen, & Jørgensen, 2009; Stelbrink et al., 2020; Van Bocxlaer & Strong, 2019). The taxonomic issues of *S. quadrata* deserve a comprehensive study but are not the topics of this study. It feeds on small algae or large plant epidermis or other organic matter, and it can usually be collected on aquatic plants or bottom sediment in a shallow area of both natural and aquaculture water bodies. The female breeds throughout the year, and the juvenile develops to sexual maturity in 1 year (Liu et al., 1979). It is gonochoristic with internal fertilization, and the brood pouch contains encapsulated eggs, and it also has no dispersive larval phase (Liu et al., 1979; Van Bocxlaer & Strong, 2019). Human use of this snail differs greatly between China and Japan. *S. quadrata* has wide adaptability to various freshwater environments, a fast growth rate, and a large reproductive capacity, and it is treated as a common food resource in China (Wen, Qin, & Huang, 2018). The average total capture production of freshwater snails per year in China is approximate 1.174 × 10^8^ kg (average capture per unit area, 12.23 kg/km^2^) (Agency of Fisheries Adminstration, Ministry of Agriculture, & Rueal Affairs of Peoples' Republic of China, 2003–2017) while that in Japan is only approximate 2.378 × 10^6^ kg (average capture per unit area, 6.29 kg/km^2^) (Ministry of Agriculture, Forestry, & Fisheries of Japan, 2019). The average capture production of freshwater snails per unit area in China is about twice the size of that in Japan. If we assume that the biomass proportion and capture intensity of *S. quadrata* in a freshwater habitat are the same in the two countries, the capture production of *S. quadrata* per unit area in China would be also about twice the size of that in Japan. However, the real situations are very different, especially food consumption of *S. quadrata* in the aquatic market, although there is no accurate data to support these ideas. People can buy this snail from the market for cooking in China, but eating viviparid snails, especially *S. quadrata*, is not popular in Japan. The much larger aquatic consumer market of *S. quadrata* in China potentially enhances the transportation of this snail (Wen et al., 2018), which highly helps to promote human‐vectored dispersal of *S. quadrata*. Thus, the different human uses that result in the different human‐vectored dispersals may influence the snail's recent gene flow to a certain extent. This influence of human transportation was mentioned in Gu, Husemann, et al. (2015), Gu, Zhang, et al. (2015), Gu, Zhou, et al. (2015) but they did not make a comparison study between different distribution areas, China and Japan, which has different human use of these snails.

It is suggested that *S. quadrata* in Japan was introduced from China, but this remains inconclusive (Hirano et al., 2015). Fossils of *S. quadrata* in China were found in the Late Pleistocene and Early Holocene sediments of 11,000–6,000 years ago (Huang, Zhu, Cai, & Tian, 2007; Wang, 1961, 1983). The oldest *S. quadrata* fossils in Japan are from the southern Kyushu, which is also about 10,000–6,000 years ago (Matsuoka unpublished), but fossil records in other parts of Japan (Honshu) are more recent about 4,000–1,000 years ago (Kurozumi, 2013). *S. quadrata* is currently the most abundant species among Japanese viviparid species, but its presence in the fossil records that are within the past 10,000 years are very rare relative to other viviparid species (Kurozumi, 2013). Thus, *S. quadrata* in Japan was likely introduced from continental East Asia before 10,000 years ago. Historically, the East China Sea region land bridge might serve as a dispersal corridor that contributed to population distribution from continental East Asia to Japan during the Pleistocene (Kameyama, Furumichi, Li, & Tseng, 2017; Saito et al., 2018; Wepfer, Guénard, & Economo, 2016; Zhang et al., 2016; Zhao et al., 2019). The land bridge has been influenced by glacial–interglacial cycles during the Pleistocene (Voris, 2000), resulting in its repeated exposure and submergence, and this played an important role in shaping the population structure across the land bridge (Zhang et al., 2016). Combining with the fossil information of *S. quadrata*, we develop one hypothesis that the migration of *S. quadrata* into Japan is likely to contain a natural process. It originally distributed in some regions of the Eurasian margin. These regions were separated from the continent and formed parts of the Japanese archipelago in geohistory, and finally, populations in these regions settled down and experienced complex evolutionary processes with a long‐term geographical isolation. Later in recent periods, associating with human communication between Japan and continental East Asia that has continued for thousands of years, *S. quadrata* may also have been subsequently introduced several times into Japan by human activity more recently. Therefore, *S. quadrata* migration from continental East Asia to Japan might contain both processes of ancient migration caused by the division of Japan from Eurasian margin and recent introduction driven by human communication. Understanding the background of the sources and introduced populations is essential to explore the differences in recent genetic patterns between source and introduced populations.

**FIGURE 1:**
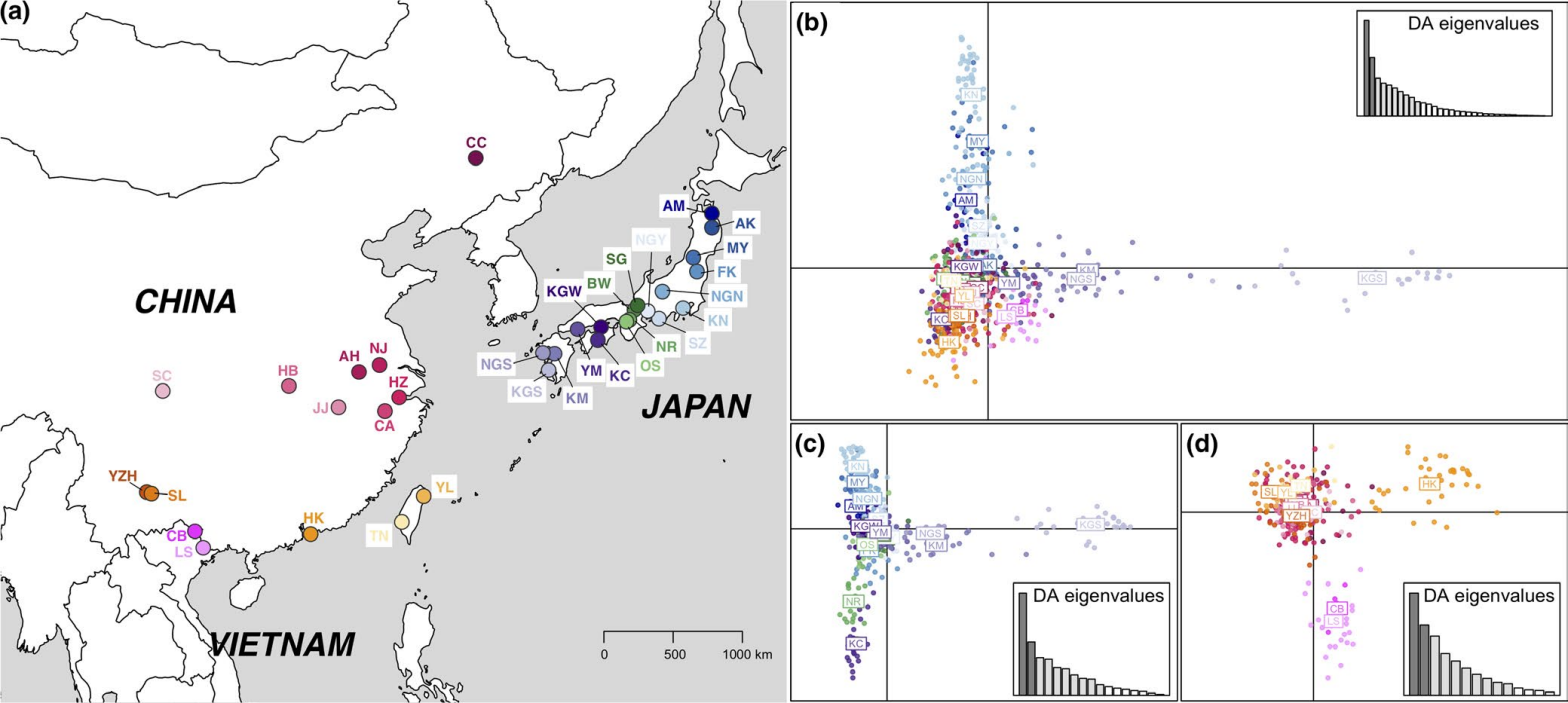
Sampling locations (a) and Population structure analyzed using DAPC (b–d) for *Sinotaia quadrata* distributed in Japan and the continental East Asia. Abbreviation letters represent location names that are also shown in Table 1. Populations in continental East Asia are highly mixed (b), and only Hong Kong, Lang Son and Cao Bang seemed different (c). Most populations in Japan are structured and form a chain from east to west, except for Kagoshima, Nagasaki, and Kumamoto in Kyushu (b, d)

Previous studies showed that the *S. quadrata* population showed a weak divergence across large geographical distances in China, suggesting that floods, human translocations, and the large population size probably weakened the genetic differentiation (Gu, Husemann, et al., 2015; Gu, Zhang, et al., 2015; Gu, Zhou, et al., 2015). However, there is a lack of data on climate and human effects to support the conclusions in these previous studies, which also did not compare the different circumstances in the various *S. quadrata* distribution areas. In this study, we tried to detect how human effects and bioclimatic variables have influenced the recent genetic divergence and we compared the genetic structure between Japan and continental East Asia, particularly China (including Taiwan island, which is geographically not on the continent but close to mainland China) and Vietnam (Figure 1). We investigated the demographic history of *S. quadrata* to detect when and how *S. quadrata* in Japan had migrated from continental East Asia. Based on the history of source and introduced populations, we subsequently compared the differences in *S. quadrata's* genetic patterns and revealed the factors that shaped the contemporary genetic divergence in the two regions. Using an example of the demographic history and population structure of a common freshwater species, we aimed to explain how ancient geological processes and recent human introduction combined with long‐term geographical isolation and short‐term modern human activity affected the biogeographical patterns in the region of East Asia.

## 2 MATERIALS AND METHODS

### 2.1 Sampling and DNA extraction

We sampled 15 sites on the continent of East Asia. We selected sampling sites that covered several representative river systems, but we did not intensively sample all over mainland China, because previous studies showed that *S. quadrata* has a weak divergence across large geographic distances in China (Gu, 2013; Gu, Husemann, et al., 2015; Gu, Zhang, et al., 2015). Two sites of Yilan and Tainan on Taiwan Island were included because, although they are not on the mainland, they are much closer to the mainland compared to the larger distance that separates Japan from the continent. We also sampled at two sites in Vietnam because they have connections through the freshwater system on the same continent (East Asia). Then, we sampled more uniformly across Japan at 18 sites, which is separated from continental East Asia by seas. Sites in Japan were sampled because possible divergence on the Japanese archipelago must be considered. Overall, we explored 33 sampling locations and obtained 778 individuals in Japan (number of populations, *N*_pop_ = 18; number of individual samples, *n* = 426), China (*N*_pop_ = 13, *n* = 326), and Vietnam (*N*_pop_ = 2, *n* = 26) (Table 1). We randomly collected samples from lakes, ponds, or water channels that were mostly around rice fields, reservoirs, parks, or villages at each site. All sites were related to human habitats, and a few samples were even bought from markets (Table 1). We identified the species directly in the field. We stored muscle tissue samples in 99% ethanol and then used a modified phenol–chloroform method, which was described by Hirano et al. (2015), to extract total genomic DNA from each sample. We calculated the geographic distance between pairwise populations using the Geographic Distance Matrix Generator (GDMG) v1.2.3 (Ersts, 2018), based on the geographic coordinates of the sampling location in each population (Table 1).

**TABLE 1:** *Sinotaia quadrata* samples (*N* = 778) used in this study

No.	Site	Label	Longitude	Latitude	*n*	*N_A_*	*H_O_*	*H_E_*	*HWE*	*Ap*
Japan							0.4919	0.6692		
1	Aomori	AM	141.32549	40.71090	13	6.833	0.5399	0.7495	***	0.07
2	Akita	AK	141.32549	39.91159	25	9.417	0.5272	0.7214	***	0.27
3	Miyagi	MY	140.06500	38.20503	21	6.583	0.4991	0.5728	***	0.11
4	Fukushima	FK	140.30995	37.41986	23	9.417	0.4837	0.7287	***	0.17
5	Nagano	NGN	137.97012	36.27872	28	7.417	0.6012	0.6512	***	0.13
6	Kanagawa	KN	139.36815	35.34148	48	6.250	0.2858	0.4508	***	0.13
7	Shizuoka	SZ	137.73195	34.74079	24	6.583	0.5600	0.6444	***	0.16
8	Nagoya	NGY	136.97387	35.17167	24	8.167	0.6013	0.7664	***	0.21
9	Shiga	SG	136.28629	35.49361	23	12.667	0.7218	0.8604	***	0.30
10	Biwako	BW	136.07092	35.24254	20	10.167	0.6717	0.8050	***	0.28
11	Nara	NR	135.73082	34.67111	22	6.917	0.6588	0.7415	***	0.10
12	Osaka	OS	135.52899	34.56866	13	10.250	0.6338	0.8457	***	0.19
13	Kagawa	KGW	133.80557	34.26782	31	10.083	0.5152	0.7264	***	0.29
14	Kochi	KC	133.59092	33.52595	21	6.167	0.4905	0.5415	***	0.07
15	Yamaguchi	YM	132.21441	34.14158	33	7.833	0.4827	0.7140	***	0.18
16	Kumamoto	KT	130.65568	32.75014	22	6.333	0.4066	0.5654	***	0.18
17	Nagasaki	NGS	129.71472	33.17973	9	4.500	0.3563	0.5897	***	0.10
18	Kagoshima	KGS	130.25028	31.82426	26	4.300	0.2282	0.3709	***	0.11
China‐Vietnam							0.6149	0.8398		
19	Changchun	CC	125.30809	43.85745	27	11.500	0.5318	0.8148	***	0.15
20	Anhui^a^	AN	117.38800	31.71570	20	14.833	0.6418	0.8905	***	0.60
21	Nanjing	NJ	118.78347	32.10670	48	20.583	0.6426	0.879	***	0.36
22	Hangzhou	HZ	120.11192	30.27164	42	20.917	0.6174	0.8780	***	0.44
23	ChunAn	CA	119.14888	29.49127	20	13.750	0.6830	0.8803	***	0.67
24	Hubei^a^	HB	112.63410	30.92060	16	14.000	0.5649	0.8952	***	0.41
25	Jiujiang	JJ	115.99929	29.70966	20	13.333	0.6254	0.8615	***	0.42
26	Sichuan	SC	30.63369	104.07721	9	7.417	0.4715	0.8098	***	0.27
27	Yangzonghai	YZH	102.99134	24.89683	32	12.833	0.6516	0.8099	***	0.30
28	Shilin	SL	103.31128	24.81002	20	8.583	0.6530	0.7504	***	0.42
29	HongKong	HK	114.11083	22.51421	37	11.667	0.5027	0.7516	***	0.23
30	Yilan	YL	121.77606	24.66726	19	8.417	0.7350	0.8582	***	0.33
31	Tainan	TN	120.29436	23.19994	10	9.988	0.6732	0.8387	***	0.11
32	Cao Bang^a^	CB	106.26256	22.66817	6	4.900	0.6433	0.7103	0.0581	0.40
33	Lang Son^a^	LS	106.82302	21.73836	26	9.250	0.4716	0.7316	***	0.29

Note

HWE indicated the test significance for deviation to Hardy–Weinberg equilibrium: *p < .05; **p < .01; ***p < .001.

^a^ Part of samples was bought from market. The approximate co‐ordinates of market samples were from the seller's information. Final co‐ordinates used for geographic distance calculation were estimated according to the centroid of all sampling sites. For each sampling site, the following are indicated: GPS coordinates (in decimal degrees), total sample size (*n*), average number of alleles (*NA*), observed (*HO*) and expected (*HE*) heterozygosities, and private allelic richness Ap(10).

### 2.2 Microsatellite genotyping

After pretesting several microsatellite markers for *S. quadrata*, which were developed by Gu et al. (2012a,2012b), we chose 12 loci for stable amplification (Table S1 in Appendix S1, deposited in http://doi.org/10.5061/dryad.h18931zgk, the same for Appendices below). In the PCR protocol, we used normal forward primers and fluorochrome‐labeled forward primers, and added a 7‐bp PIG‐tail (5′‐GTTTCTT‐3′) to the 5′ end of reverse primers to enhance genotype specificity (Brownstein, Carpten, & Smith, 1996). Each PCR amplification reaction was performed in a total volume of 2 μl, which included the following: 1 μl of 2× PCR MasterMix (Qiagen), 0.1 μl of forward primer mixture that was mixed with four different fluorochrome‐labeled forward primers (0.125 μM of each) together with the corresponding normal forward primer (1.875 μM), 0.1 μl of corresponding reverse primer mixture that had 5′ PIG‐tail (2 μM), 0.8 μl of ddH_2_O, and 0.3 μl of genomic DNA (dried in advance, and the concentration was not quantified). Thermocycling conditions were as follows: 95°C for 5 min + 30 × (95°C for 30 s, 60°C for 90 s, 72°C for 30 s) + 60°C for 30 min (Bio‐Rad T100™, Thermal Cycler). PCR products were genotyped using an ABI 3130 genetic analyzer (Applied Biosystems), and allelic sizes were scored by GeneMapper v4.0 (Applied Biosystems) using an internal size standard (GeneScan LIZ500, Applied Biosystems). Questionable specimens and poor amplification with hard‐to‐read genotypes were re‐extracted and reamplified. Genotypes that have less than 20% of missing data were considered for all analyses.

### 2.3 Population genetics

We estimated the frequency of null alleles for each locus and population with a microsatellite dataset using the expectation maximation algorithm that was described by Dempster, Laird, and Rubin (1977) and redesigned in the program FreeNA (mentioned below) by Chapuis and Estoup (2007). It is a correction of conventional methods and provides a better estimate of genetic distance with harboring null alleles. Because null alleles are universal and complex in the microsatellite analysis, species with a large effective population size usually has a relatively high frequency of null alleles (Chapuis & Estoup, 2007), such as reports of insects (Chapuis, Loiseau, Michalakis, Lecoq, & Estoup, 2005; Lehmann et al., 1997; Meglecz et al., 2004) and mollusks (Astanei, Gosling, Wilson, & Powell, 2005; Li, Hubert, Bucklin, Ribes, & Hedgecock, 2003). If the null allele frequency was larger than 0.2 in population genetic studies, it was better to eliminate this locus or use newly designed primers (Wen et al., 2013). In our analysis, the percentage of the null allele frequency that was larger than 0.2 ranged from 0 (TXH65) to 29% (TXH30) for each locus in all the populations (Table S2 in Appendix S1). However, for different populations, the null allele frequency of the same locus showed a large variation (i.e., TXH30), which suggested that the occurrence of null alleles was randomized at the intraspecies level (Wen et al., 2013). Considering the potential Wahlund effect (i.e. mixture of population samples from multiple sources or locations), it was difficult to decide which locus should be dropped. Therefore, we followed Dempster's method (1977) to estimate the global *F_ST_* for each locus and the pairwise population *F_ST_* using FreeNA (Chapuis & Estoup, 2007). By testing the Pearson's correlation between the pairwise population *F_ST_* (harboring null alleles) and the pairwise population *G_ST__H*, which was estimated with null alleles included (Philip, 2005) (total: *r* = 0.8416, *p* < .0001; Japan: *r* = 0.8696, *p* < .0001; continental East Asia: *r* = 0.9150, *p* < .0001), we found that null alleles had no significant impact on subsequent analyses. Therefore, we reported subsequent results using the full microsatellite dataset. We estimated the average number of alleles (*N_A_*) and the observed (*H_O_*) and expected (*H_E_*) heterozygosities, and we tested the analysis of molecular variances (AMOVA) using Arlequin v3.5 (Excoffier & Lischer, 2010). We estimated the linkage disequilibrium (LD) between loci pairs for the total dataset and for each population, and tested the global Hardy‐Weinberg Equilibrium (HWE) using a Markov chain algorithm with 10,000 dememorizations, 1,000 batches, and 10,000 iterations per batch using GENEPOP v4.2 (Raymond & Rousset, 1995; Rousset, 2008). We computed the private allelic richness of each locus within each population using HP‐Rare (Kalinowski, 2004, 2005).

### 2.4 Population structure

We performed discriminant analysis of principal component (DAPC) using the microsatellite data to detect the overall genetic structure, and also that in Japan and continental East Asia using the adegenet v2.1.0 package in R (Jombart, 2008; Jombart, Devillard, & Balloux, 2010). We first pooled individuals without previous information to determine the optimal numbers for the groups by checking the lowest Bayesian information criterion (BIC) (Jombart et al., 2010). We covered several clusters *K* from 1 to 30, which was designated as twice the optimal simulation *K*, and maintained consistency in each analysis to allow comparison of the results (Jombart et al., 2010). Then, we performed DAPC containing the previous information from each individual and used the function *optim.a.score* to select the optimal number of retained principal components (PCs). We also identified individual assignments using STRUCTURE v2.3.4 (Falush, Stephens, & Pritchard, 2003; Pritchard, Stephens, & Donnelly, 2000). In the first round, we analyzed the structure of the overall populations by choosing the same range of clusters *K* (1 − 30) as were used in DAPC. In the second round, we analyzed the structure of Japan and continental East Asia separately by choosing the range of *K* that was equal to the number of populations in each group. All independent runs were computed using an admixture model, allele frequencies that were correlated with a length of 10,000 burn‐in period, 50,000 Markov chain Monte Carlo replications after burn‐in, and ten iterations for each run for *K*. The optimal number for *K* was selected using Structure Harvester Web v0.6.94, according to the highest value of Evanno's delta *K* (Earl & Vonholdt, 2012; Evanno, Regnaut, & Goudet, 2005). Because different values of *K* might reflect different demographic information, we also interpreted the structure patterns from “suboptimal” *K*, which was the other peaks of delta *K* (Meirmans, 2015). Structure patterns were summarized for the optimal and suboptimal *K* based on merged iteration runs using Pophelper Web App v1.0.10 (Francis, 2017).

### 2.5 Isolation by distance and gene flow

To provide a better explanation for the genetic diversity (Alcala, Goudet, & Vuilleumier, 2014; Leng & Zhang, 2011), we measured the genetic distance of both *G_ST__H* (Philip, 2005) and *D_J_* (Jost, 2008) between pairwise populations overall, in Japan, and in continental East Asia using the FinePop v1.5.1 package in R (Kitada, Nakamichi, & Kishino, 2017). Then, we computed the natural logarithm value of the geographical distance and tested its correlation with the genetic distance (*G_ST__H and D_J_*, respectively) using the Mantel tests with 10,000 permutations and a linear regression model to estimate the isolation by distance at each group level. We detected the local historical gene flow within Japan and continental East Asia using the maximum likelihood algorithm with Migrate 3.6.11 (Beerli, 2006). We estimated *θ* = 4*Nm_μ_* (*N*, the effective population size; *m_μ_*, the mutation rate per locus per generation) and *M = m/m_μ_* (*m*, the migration rate per generation), and calculated the effective number of migrants (4*Nm = θ* × *M*). We ran ten short chains (10,000 trees), two long chains (100,000 trees), burn‐in 10,000 initial trees, and combined long chains for estimates. The starting values for *θ* and *M* were estimated from the *F_ST_* values. All individuals were included, and three independent analyses were performed.

### 2.6 Human and bioclimatic effects on genetic divergence

We collected data from human effect variables at each location from the Socioeconomic Data and Applications Center (SEDAC) using NASA's Earth Observation system Data and Information System (EOSDIS) (https://sedac.ciesin.columbia.edu). Human population density (PopD) data were collected from the Population Density, v4.11 (2015) with a 2.5 arc‐minute resolution (Center for International Earth Science Information Network CCU, 2018). Human footprint (HmFp) data were collected from the Human Footprint, 2018 Release (2009) with a spatial resolution of approximately 1 km (Venter et al., 2016, 2018). Human net migration (NetM) data were collected from the Global Estimated Net Migration Grid by Decades v1 (1990−2000) with a half‐degree and 30 arc‐second grid cell resolution(de Sherbinin et al., 2012; de Sherbinin et al., 2015). Urban expansion (UbEx) data were collected from the Development Threat Index, v1 (2015) with a 50 square‐kilometer grid cell resolution (Oakleaf et al., 2015; Oakleaf et al., 2019). To measure the impact of variables more accurately, we specified buffer radii of 1, 5, 10, 20, and 50 km around each sampling location to extract PopD and HmFp data, and specified buffer radii of 20 and 50 km around each sampling location to extract NetM and UbEx data using the raster v2.9–5 package in R (Hijmans, 2019). We limited the buffer radius up to 50 km to avoid too much data overlap for locations that were close together (Figure S1 in Appendix S2). We used the absolute value of NetM in our analysis because both in migrants (positive) and out migrants (negative) in NetM reflected human disturbances at these locations. We also accessed 19 bioclimatic variables (from BIO1 to BIO19) of temperature and precipitation data from WorldClim (http://worldclim.org) (resolution, 10 min of a degree) using the function getData in the raster v2.9–5 package in R (Hijmans, 2019). The meaning of each variable was listed in Table S3 (Appendix S1). All data on human and bioclimatic variables are shown in Appendix S3.

We performed a random forest classification analysis to identify variables that were important for explaining population divergence (*F_IS_*) using all the following variables: human effect, bioclimate, longitude, and latitude. All predictor variables were standardized and tested using the randomForest package in R (Liaw & Wiener, 2002). We selected the number of parameters that were sampled for each node split (mtry) using the tuneRF function with 10,000 trees. The optimal mtry parameters were then used for classification. We ran a classification with random forest by setting 10,000 trees to grow and all other parameters to the default. We measured the relative importance of each predictor variable using the mean decrease in accuracy (MDA) that was averaged over all trees. MDA values represent how the accuracy of random forest decreases when the predictor variable is excluded, and thus, higher MDA values indicate greater importance of the variables in the model and negative MDA values indicate that the incorporation of variables reduces the model accuracy. We used all the predictor variables with positive MDA values as exploratory variables, and we used the allele frequency of each population that was computed based on Meirmans (2015) as dependent variables for redundancy analysis (RDA) within the overall population, and in Japan and continental East Asia. Thus, the RDA can tell us how these variables correlate with population genetics. We performed a forward model choice to test the significant variables for RDA (Table S4 in Appendix S1) using the ordistep function in the vegan package in R (Oksanen, Blanchet, & Friendly, 2019). We assessed the significance of RDA and evaluated the contribution of each component by testing the analyses of variance (ANOVA) using the global and axis methods with 10,000 permutations.

### 2.7 Model checking for population history

We inferred the population history across East Asia using the Approximate Bayesian Computations (ABC) method with microsatellite loci data to check population history models. We estimated the population divergence, admixtures, and population size changes in the demographic population history using DIYABC 2.1.0 (Cornuet et al., 2014; Cornuet, Ravigné, & Estoup, 2010). We defined demographic scenarios based on our results of the genetic structure, which was inferred by microsatellite loci (see the Section 3.1 & 3.2) and the current evolutionary knowledge about *S. quadrata*. Populations were uniform across continental East Asia, and thus, all populations in continental East Asia were grouped and defined as CN. For Japan, populations in Kyushu (one of the four islands in Japan) were separated from others and were defined as the JPK group. The other populations were structured corresponding to the geographical patterns and were grouped into two parts: JP1 and JP2. The JP1 lineage was consistent with the usual area division in Japan called Tohoku (meaning Northeast) and Kanto (meaning East). The JP2 lineage was consistent with the usual area division in Japan, which is called Kansai (meaning West) and Shikoku (one of the four main islands of Japan), and had admixtures with populations on continental East Asia (CN). Populations in the continental East Asia (CN) were considered to be the source (Hirano et al., 2015; Saito et al., 2018). Kyushu has the shortest distance from the continent and it was attached to the continent in ancient times (Kitamura & Kimoto, 2006; Martin, 2011; Otofuji, Matsuda, & Nohda, 1985; Takenaka & Tojo, 2019), suggesting that the earliest introduction from the continent might have happened here. Additionally, the historical population size changes were assumed to be an expansion model because *S. quadrata* is currently distributed widely. Therefore, we defined 12 demographic scenarios under these assumptions (see details in Section 3.5).

To balance the sample size of lineages and to determine the computing time, we randomly choose 60 individuals in lineages from JP1, JP2, and CN, and retained all individuals (*n* = 57) in JPK (Appendix S4). We defined the historical parameters by performing the pilot run with wide extensive parameters and all summary statistic values (Table S5 in Appendix S1). Overall, we computed seven summary statistics for the microsatellite group (mean number of alleles, mean genetic diversity, mean size variance for one sample, mean number of alleles, *F_ST_*, classification index, and (*dμ*)^2^ distance for two samples). We set all of the microsatellite mutation models to the default settings. Then, we compared the different scenarios by computing their relative posterior probabilities by checking linear discriminant analysis on summary statistics using a logistic regression method from 10% of the simulated data sets that most closely resembled the observed data set. Under the most likely scenario, as determined by the logit transformation of parameters, we estimated the posterior parameter distribution in 10% of the simulated data sets that most closely resembled the observed data set. Then, we used the option of model checking with principal component analysis to evaluate how well the posterior predictive distribution of the model fit the observed data for the best scenario. Finally, using linear discriminant analysis on summary statistics, we evaluated the confidence in the scenario choice by computing the scenario‐specific prior‐based error using historical parameters that were drawn from the prior distribution and 1% of simulated data sets that most closely resembled the observed data set using the logistic approach with other parameters set to default settings.

## 3 RESULTS

### 3.1 Population genetics

The average observed heterozygosity of continental East Asia (0.6149) was higher than that of Japan (0.4919) (Table 1). *H_O_* ranged from 0.4715 (Sichuan) to 0.7350 (Yilan) in China, from 0.2282 (Kagoshima) to 0.6588 (Nara) in Japan, and from 0.4716 to 0.6433 in Vietnam (Table 1). Almost all populations deviated from the Hardy–Weinberg equilibrium (HWE), except for Cao Bang (with only six samples). Private alleles were present within all populations but with a relatively higher frequency in China. In the overall dataset, nine of 60 loci pairs showed significant linkage disequilibrium (Table S6 in Appendix S1), but 3.4% of all loci pairs were detected as being linked when each population was tested separately. After the populations were divided into groups of Japan and continental East Asia, AMOVA detected 3.85% variation between Japan and continental East Asia, and a 17.32% variation among populations within groups, which suggested that a significant genetic differentiation exists between groups (AMOVA *F_CT_* = 0.0385, *p* < .001) and among populations in different groups (*F_ST_* = 0.2118, *p* < .001; Table 2). The fixation index *F_IS_* for each population was estimated and used for multivariable analysis of population genetics (Appendix S3). The range of pairwise genetic distance was as follows: i) *G_ST__H* is from 0.1369 (Shiga‐Biwako pair) to 0.9632 (Kagoshima‐Kanagawa pair); and ii) *D_J_* is from 0.1258 (Shiga‐Biwako pair) to 0.9372 (Kagoshima‐Shizuoka pair).

**TABLE 2:** AMOVA analysis for total populations and groups

Source of variance	Sum of squares	Variance of components	Percentage variation (%)
AMOVA results of all populations			
Among populations	362.454	0.222	19.670
Within populations	1,382.127	0.908	80.330
Total	1744.582	1.130	
*F*_ST_ = 0.1967***			
AMOVA results of populations in two groups (Japan, continental East Asia)			
Among groups	46.294	0.044	3.850
Among populations within groups	316.160	0.199	17.320
Within populations	1,382.127	0.908	78.830
Total	1744.582	1.151	
*F*_ST_ = 0.2118***, *F*_SC_ = 0.1802***, *F*_CT_ = 0.0385***			

*** *p* < .001.

### 3.2 Population structure

The DAPC with previous information retained 58, 53, and 53 PCs overall and for Japan and continental East Asia, respectively (Figure S2 in Appendix S2). The results showed that populations were mixed and showed no obvious edge and structure in continental East Asia (Figure 1b), while Hong Kong and Vietnam (Lang Son and Cao Bang) seemed to be different (Figure 1d). Most populations in Japan were highly structured and formed a chain from the northeast (e.g. Akita) to the southwest (e.g. Kochi) (Figure 1c). However, Kagoshima, Nagasaki, and Kumamoto on Kyushu island were separated from the main chain with a few overlaps on the DAPC axis 1 (Figure 1b,c). Clusters driven by Kochi, Nara, Shiga, Biwako, and Osaka in Japan were partially overlapped with populations in continental East Asia (Figure 1b). According to the lowest BIC score, DAPC without previous information on the populations identified 16, 13, and six clusters overall, and for Japan and continental East Asia, respectively. Predefined populations in Japan were obviously clustered (e.g. Kagoshima), while those in continental East Asia showed no obvious clusters, which was consistent with DAPC results that were obtained using previous information (Figure S3 in Appendix S2).

**FIGURE 2:**
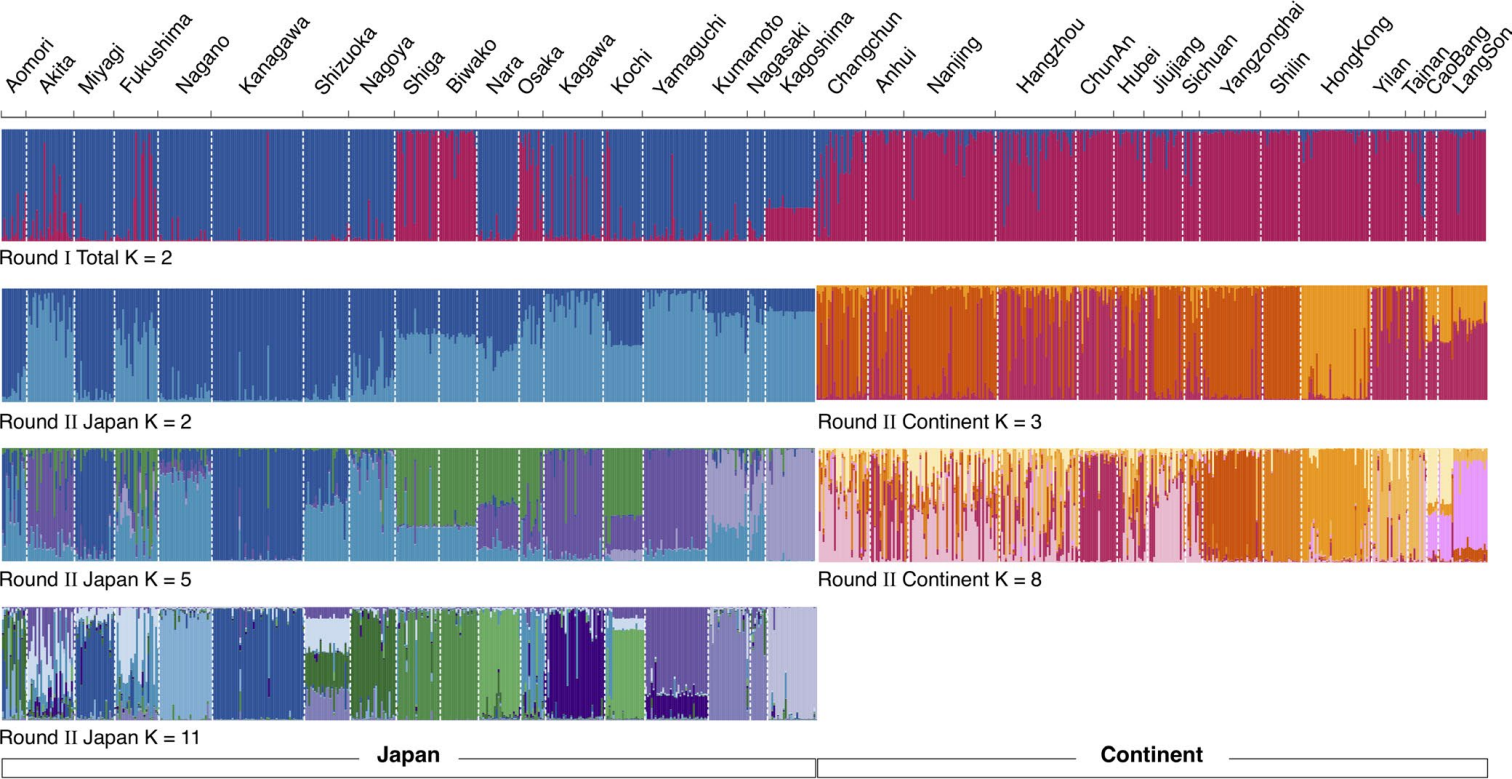
Population structure analyzed using Structure. Japan and continental East Asia are split associating with their geography and have some admixtures (Round I). Structure results suggested that Japan is highly structured according to geographic barriers, while continental East Asia is highly mixed (Round II). Blue colors represent structured clusters in Japan. Red colors represent structured clusters in continental East Asia. Clusters that are consistent with corresponding populations are colored based on Figure 1

Structure analyses identified two genetic clusters (optimal *K* = 2) for the overall populations (Figure 2). There was a split between Japan and continental East Asia, suggesting that structured populations corresponded to previous geographical locations among the two regions. Populations in continental East Asia were clustered together and had admixtures with Shiga, Biwako, Osaka, and Kagoshima in Japan, which was consistent with the DAPC results. Additionally, results of round II analysis focusing on each group showed two clusters (optimal *K* = 2) in the Japan group and three clusters (optimal *K* = 3) in the continental East Asia group. Structures illustrated by the suboptimal *K* values (Japan: *K* = 5, *K* = 11; continental East Asia: *K* = 8) in round II (Figure S4 in Appendix S2) indicated that Japan had stronger structures than continental East Asia (Figure 2, Round II). The clusters in the Japan group were generally divergent in the different geographical areas of northeast, middle, and southwest Japan (Figure 1). The clusters in the continental East Asia group showed that populations were genetically mixed, except for some special clusters that were driven by Hong Kong and Vietnam (Cao Bang and Lang Son), which was similar to the DAPC results. Additionally, population genetics in mainland China were uniform despite of the long geographic distance (e.g., Changchun, Nanjing, Yangzonghai, and Shilin) (Figure 2, Round II).

### 3.3 Isolation by distance and gene flow

**FIGURE 3:**
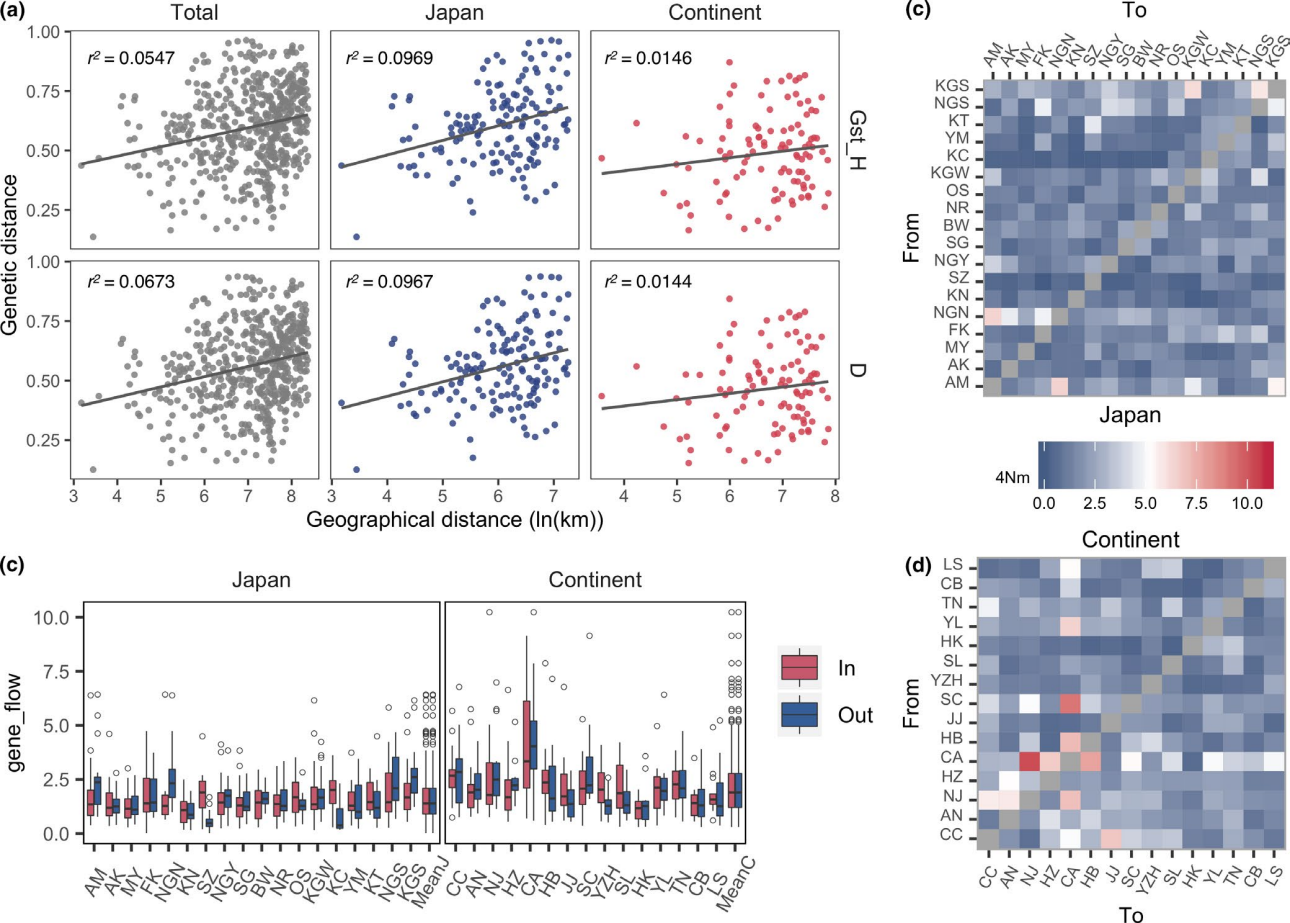
Isolation by distance and gene flow in Japan and continental East Asia. Linear regression between natural logistic value of geographical distance and genetic distance assessing by *G_ST__H* and *D_J_* showed higher significance overall and in Japan compared to that in the continental East Asia (a). *r^2^* represents the linear regression value for each model. Gene flow values (*4 Nm*) within the continental East Asia are relatively higher than that within Japan (b). In Japan, gene flow from Kyushu to other parts is a little bit higher (c). In the continental East Asia, gene flow from the east part (e.g. NJ, CA, HB) to other parts is relatively higher (d). Population abbreviations are listed in Table 1

The correlation between genetic and geographic distance matrices using the Mantel test showed a significant correlation among all the populations and the Japan group, but no significant correlation with the continental East Asia group (Table 3). Linear regression between the genetic distance and geographic distance also supported the results that populations in the Japan group were more significantly divided by geographic distance than populations in continental East Asia (Table 3 and Figure 3a). The historical gene flow (4*Nm*) between population pairs was higher among regions of continental East Asia than regions in Japan (Figure 3b). In Japan, gene flow from the Kyushu area (e.g., Kagoshima, Nagasaki) to other parts was relatively higher (Figure 3c). In continental East Asia, gene flow from the eastern part of China (e.g. Nanjing, Hangzhou, ChunAn) to other parts was relatively higher (Figure 3d).

**TABLE 3:** Correlations between genetic distance (*G_ST__H*, *D_J_*) and geographic distance (*dist_Geo_*) of pairwise populations

Correlation	Total	Japan	China–Vietnam
Mantel test			
*G_ST__H* ~ *dist_Geo_*			
*r*	0.2358	0.3159	0.1390
*p*	0.0007***	0.0025**	0.1044
*D_J_* ~ *dist_Geo_*			
*r*	0.2610	0.3156	0.1387
*p*	0.0003***	0.0013**	0.1007
Linear regression			
*G_ST__H* ~ *dist_Geo_*			
*r^2^*	0.0547	0.0969	0.0146
*p*	8.3e‐15***	1.6e‐08***	0.0440*
*D_J_* ~ *dist_Geo_*			
*r^2^*	0.0673	0.0967	0.0144
*p*	2.2e‐16***	1.7e‐08***	0.0452*

* *p* < .05, **p < .01, ***p < .001.

### 3.4 Human and environmental correlates of genetic divergence

**FIGURE 4:**
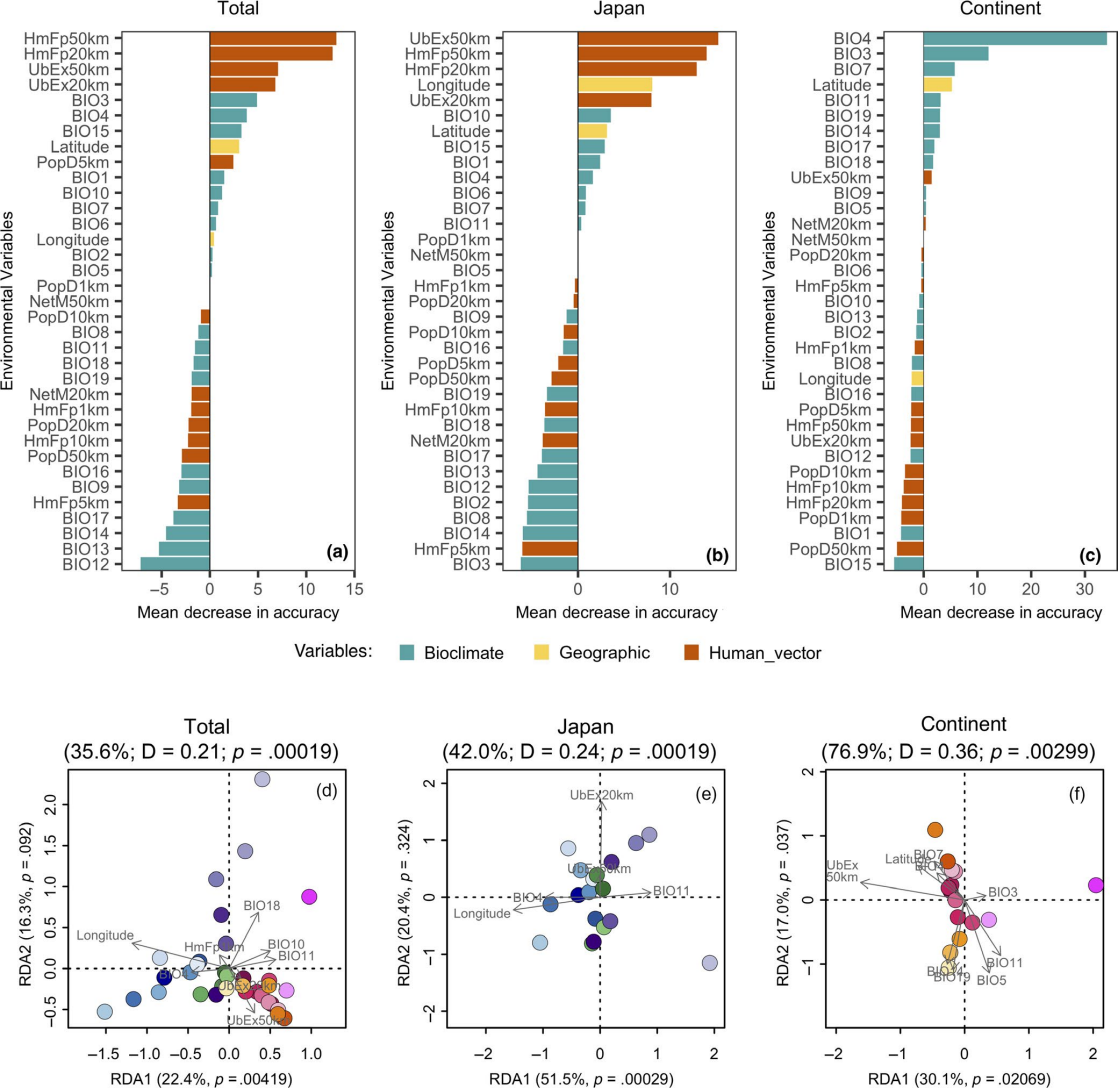
The importance of the variables for explaining the population divergence were tested using random forest classification (a–c) and redundant analysis (RDA) based on population allele frequencies and significant variables (d–f). Urban expression is one of the most significant variables that influences population genetics in the continental East Asia (d, f), although it is not very important (c). Longitude is a very important variable and significantly influences population genetics in Japan (b, d, e), while urban expansion is important, but it is not significant (b, e). The explanations of variables are listed in Table S3 (Appendix S1)

To illustrate the relationship between human disturbance of the environment and population divergence, and to reveal the extent of the differences in the aspects of human effects between Japan and continental East Asia, we investigated the relationships between population divergence (*F_IS_*) and bioclimatic variables, geographical longitude and latitude, and major variables of human intervention in the environment (human population density (Center for International Earth Science Information Network, 2018), footprint (Venter et al., 2016, 2018), migration (de Sherbinin et al., 2012, 2015), and urban expansion (Oakleaf et al., 2015, 2019)) (see the Methods section). We identified top variables that are important for explaining variation in population divergence at each level (Figure 4a–c). Top variables with a positive mean MDA were collapsed into a RDA compared to the microsatellite allele frequency of each population (Figure 4d–f). In the overall populations, human intervention and disturbance (human pressure, density, and urban expansion) were important for explaining genetic divergence (Figure 4a), among which longitude and temperature (e.g. BIO11) significantly influenced divergence in Japan (Figure 4d,e), whereas urban expansion (UbEx 20 km, UbEx 50 km) and temperature (e.g. BIO10, BIO11) significantly affected divergence on continental East Asia. Urban expansion variables were also important for explaining variations in Japan (Figure 4b), but they had no significance on the RDA2 axis (Figure 4e). In the continental East Asia group, many bioclimatic variables and a few human effect variables were important (Figure 4c), reflecting that there were similar levels of human disturbance for different populations of *S. quadrata* in continental East Asia, which make the high admixture of population genetics (Gu, Husemann, et al., 2015; Gu, Zhang, et al., 2015; Gu, Zhou, et al., 2015; and our results) and probably reduce the importance of some anthropic variables. But the RDA results revealed that most populations were on the negative side of the RDA 1 axis, suggesting that population genetics were widely and significantly influenced by some human interventions in the continent. Variances in the continent that were shown on the RDA2 axis were mainly caused by the factors of temperature (e.g. BIO3, BIO5, BIO11) and precipitation (BIO14, BIO19) (Figure 4f).

### 3.5 Demographic history and introduction

**FIGURE 5:**
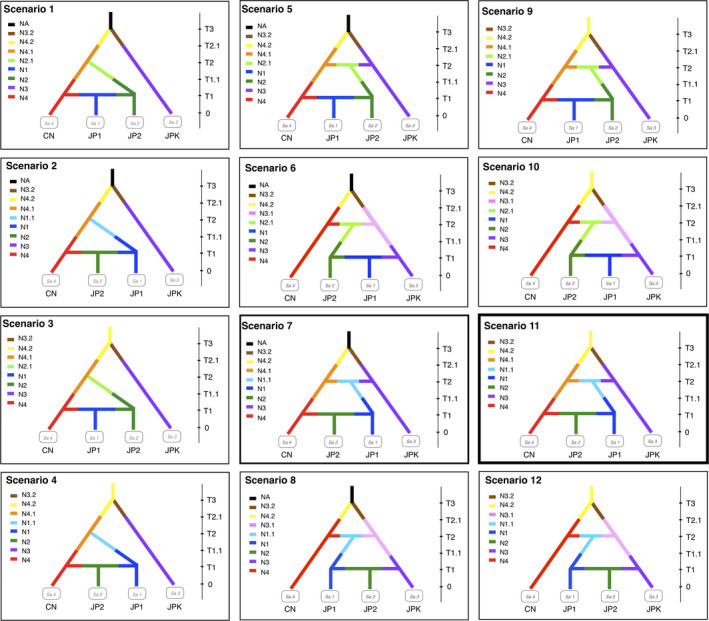
Scenarios defined based on previous of genetic structures and evolutionary backgrounds for analyzing demographic history in DIYABC. Results show that scenario 11 was the best model and scenario 7 was the second model. T represents the divergence time of lineages. N represents effective population size of each population in each period. CN represents linage of populations in continental East Asia. JP1 represents linage of populations in Tohoku and Kanto. JP2 represents linage of populations in Kansai and Shikoku. JPK represents linage of populations in Kyushu

**FIGURE 6:**
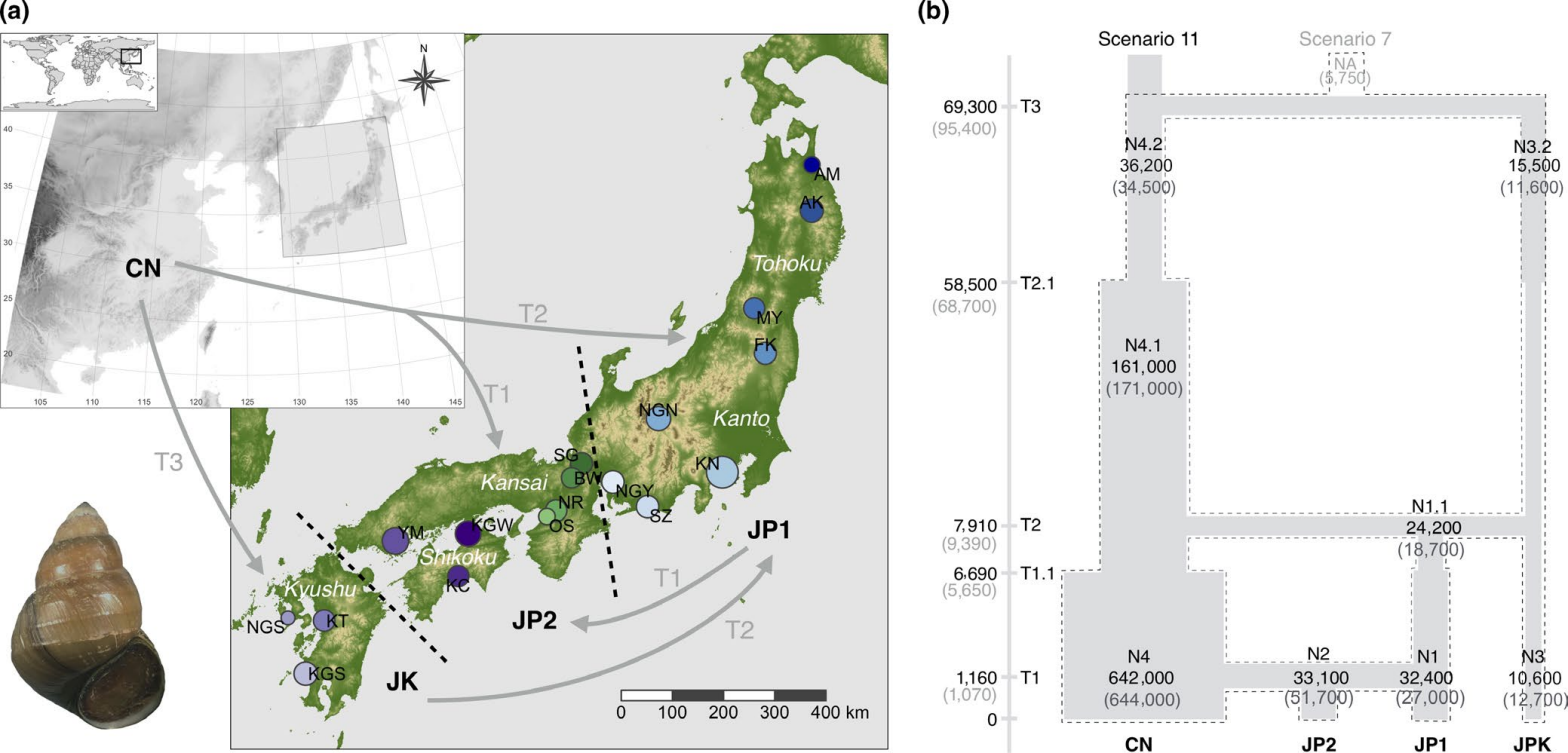
Demographic history and parameters of *Sinotaia quadrata* introduced from continental East Asia. (a) Arrows and small maps represent the historical introduction approaches of *S. quadrata* from continental East Asia (CN) to Japan based on the best model (scenario 11, see the Section 3.5). The first introduction happened in Kyushu (JPK). Then, eastern Japan (JP1) was formed by admixture of CN and JPK, and western Japan (JP2) was formed by admixture of CN and JP1. (b) The best model scenario 11 indicates that Kyushu was separated from the continent, which is shown as the solid gray box. The second model scenario 7 indicates that Kyushu and the continent were separated from an unknown ancestor NA, which is shown as the dotted box. Demographic parameters of scenarios 11 (black) and 7 (gray) are shown in the parentheses

We tested the divergence times, admixtures, and population size changes by testing 12 different demographic scenarios (Figure 5) using the ABC method (see the Methods section). The results showed that scenario 11 had the highest posterior probability and scenario 7 had a close probability to scenario 11 (Table S7 in Appendix S1 and Figure S5 in Appendix S2). Scenario 11 indicated that populations from Kyushu (JPK) were divided from the eastern Asian continent (CN) at T3, while scenario 7 indicated that CN and JPK were sister groups that had divided from the same ancestral population (NA) at T3, and the other demography of the two scenarios after T3 were the same (Figure 5). The posterior demographic parameters in scenarios 11 and 7 are shown in Table S8 (Appendix S1), Figures S6 and S7 (Appendix S2). In scenario 11, JPK was divided from CN 6.93 × 10^4^ generations ago (median value) at time T3 (95% CI: 2.11 × 10^4^–1.81 × 10^5^). Eastern Japan (JP1, see the Section 2.7) was mixed with CN and JPK and diverged at 7.91 × 10^3^ generations (median value) ago at time T2 (95% CI: 1.83 × 10^3^–3.87 × 10^4^). Western Japan (JP2, see Section 2) was mixed with CN and JP1 and diverged at 1.16 × 10^3^ generations ago (median value) at time T1 (95% CI: 74.5–7.81 × 10^3^). Expansions for the corresponding populations occurred at T2.1 (median, 5.85 × 10^4^; 95% CI, 2.27 × 10^4^–9.68 × 10^4^) and T1.1 (median, 6.69 × 10^3^; 95% CI: 1.21 × 10^3^–1.86 × 10^4^) (Figure 6b). Previous studies showed that the *S. quadrata* sexual maturation is likely to take 1 year, with iteroparous reproduction taking several years thereafter (Liu et al., 1979; Van Bocxlaer & Strong, 2019). Therefore, we inferred that the earliest introduction from CN to JPK happened approximately 70,000 years ago, and that JP1 was separated from CN and JPK approximately 8,000 years ago, while JP2 was separated from CN and JP1 approximately 1,200 years ago (Figure 6b). The PCA results from scenario 11 are shown in Figure S8 (Appendix S2). The type I error (the probability that a true scenario is rejected) and type II error (probability of false‐positive) for scenario 11 are shown in Table S9 (Appendix S1). The nonconflicting scenarios 11 and 7 indicated that *S. quadrata* in Kyushu have unique genetic properties and that they probably migrated from China around 70,000 years ago. Subsequently, the populations of Kyushu spread eastward and mixed with *S. quadrata* from China beginning 8,000 years ago.

## 4 DISCUSSION

### 4.1 Demographic history and ancient migration

The ancient origin and historical migration of species from eastern Asia to the Japanese archipelago have included various taxa, such as plants (Aoki et al., 2019; Deng, Jiang, Hipp, Manos, & Hahn, 2018; Xiang, Erst, Xiang, Jabbour, & Wang, 2018), mammals (Kinoshita et al., 2019), insects (Jang‐Liaw et al., 2019; Takenaka & Tojo, 2019), freshwater fish (Matsumoto et al., 2010; Saitoh, Chen, & Mayden, 2010), and freshwater snails (Saito et al., 2018), showing that agreement with geohistory of the Japanese archipelago and its connections with continental East Asia are combined to affect the biogeography. Most parts of the Japanese archipelago were attached to the eastern margin of the Eurasian continent until about 20 million years ago (Ma) (Martin, 2011; Otofuji et al., 1985; Takenaka & Tojo, 2019). The Sea of Japan opened up and Japan was separated as an island archipelago until about 15 Ma (Martin, 2011; Otofuji et al., 1985; Takenaka & Tojo, 2019) and the southwestern part of Japan was attaching to the margin of the continent until about 2 Ma (Kitamura & Kimoto, 2006). The best model of demographic history (scenario 11) in our results also suggests that *S. quadrata* in Japan migrated from continental East Asia at about 70,000 years ago (T3). This period is consistent with the hypothesis of an East China Sea land bridge as a “dispersal corridor” (Kameyama et al., 2017; Saito et al., 2018; Wepfer et al., 2016; Zhang et al., 2016; Zhao et al., 2019). During the Last Glacial Maximum (about 22,000 years ago), wide extensions of the continental shelf across the East China Sea were assumed to have been exposed and formed a land bridge, providing a corridor for species or population distribution and allowing migration of many species from continental East Asia to Japan (Lambeck, Esat, & Potter, 2002; Zhang et al., 2016). The Sea of Japan and the sea of northwest Kyushu became brackish during the last glacial period (70,000–10,000 years ago) because surface water in these areas was replaced by freshwater that was brought from the Yellow River and Yangtze River (Oba & Tanimura, 2012). Therefore, the natural ancient migration of *S. quadrata* was most likely to have occurred during this period and the first migration was shown to occur in Kyushu. Human introduction of *S. quadrata* during this period is unlikely because the first human arrival to Japan is around 40,000–30,000 years ago (Fujita et al., 2016).

However, our results do not show solid evidence to exclude the second best model (scenario 7), which indicated that populations in Kyushu had the same unknown ancestors as those in continental East Asia. Except for the origin of the Kyushu populations, scenarios 7 and 11 illustrate the identical process for populations in the other parts of the Japanese archipelago (Figure 5). Our results suggest that the first *S. quadrata* migration to Japan (Kyushu) probably contains both migration from the continent after it had geographically separated (best model of scenario 11) and ancestral divergences from the unknown ancestors before it had geographically separated (alternative model of scenario 7). This would be illustrated by the independent origins of the Japanese archipelago that originates in southwestern Japan, which is related to the coast of mainland China, but the origins of northeastern Japan are related to coast of eastern of Siberia (Otofuji et al., 1985; Tojo et al., 2017). Another viviparid species *Cipangpaludina chinensis* is likely also native to the Kyushu region, indicating its special origins during the geological ancient periods (Hirano, Saito, Tsunamoto, Koseki, Ye, et al., 2019). Further studies are needed to examine the details of the initial process.

### 4.2 Recent introduction, population admixture, and isolation

Both scenarios 11 and 7 showed the same process of divergence and admixture in the other part of Japan after populations settled in Kyushu. Populations from the continent migrated to eastern Japan approximately 8,000 years ago (T2) and mixed with some populations that migrated from Kyushu. Then, the migration continued from the continent to western Japan approximately 1,200 years ago (T1), and admixture with some populations that migrated from eastern Japan occurred (Figure 6a). Populations on Honshu Island genetically originated from the eastern Asian continent and also had some admixture with Kyushu. Within Honshu, the lineage in western Japan (JP2) was derived from the lineage in eastern Japan (JP1) that mixed with the lineage on the continent (CN) (Figure 6b). Thus, the historical patterns of *S. quadrata* spread throughout Japan are not simply a spread from west to east, but are a multiple processes of migrations and admixtures. These episodes of population spreading and mixing within Japan and migration from the continent occurred beginning 8,000 years ago are most likely to be caused by human movements with agriculture expansion (Bellwood, 2005; Diamond & Bellwood, 2003). In this period, the early paddy rice domesticates are thought to have moved from continental East Asia to Japan (Fuller et al., 2010; Kovach, Sweeney, & McCouch, 2007). The introduction of paddy rice cultivation during this period probably caused migration of the freshwater snails, because paddy and the irrigation system of agriculture are typical habitats for viviparids (e. g. Benson, 1842; Liu et al., 1979; Yen, 1937). Another episode of migration and admixture that occurred about 1,200 years ago corresponds to the timing of a rapid increase in trade between Japan and the continent (Hanihara, 1993; Omoto & Saitou, 1997). Migration of a large freshwater snail species such as *S. quadrata* from the continent to Japan did not likely occur after 8,000 years ago by natural processes because the Sea of Japan had expanded and it became an effective barrier for migration. This suggests that the recent migration from the continent to Japan that occurred between 8,000 years ago and 1,200 years ago was artificial introduction.

Higher genetic structure found in the contemporary populations in Japan suggests that these lineages from different regions of Japan were isolated after their introduction to the area. In particular, Kyushu is separated from the other regions (Figure 1b, Figure 1c). Kyushu is a region with a long volcanic history in the southwest area of the Japan archipelago (Kamata & Kodama, 1999). For instance, the eruption of Tsurumi volcano in the northeast part of Kyushu started at 60,000 years ago and continued to present during the past 30,000 years for multiple times (Fujisawa, Okuno, Nakamura, & Kobayashi, 2002). The volcanic activities of volcanoes at southern Kyushu were traced back to about 6,500 years ago (Ui & Fukuyama, 1972). Multiple volcanic eruptions might have caused *S. quadrata* to undergo repeated colonization–extirpation processes, which caused periodic losses in genetic diversity and multiple founder effects. This is consistent with the above results, which showed that the population size of Kyushu historically decreased (Figure 6). Although populations in other parts of Japan probably received ongoing introductions from continental East Asia throughout history (T2 and T3), the long‐term isolation caused by various geographical patterns would be the main reason why contemporary populations in Japan show significant isolation by distance (IBD) and a high genetic structure. However, populations in the larger geographical range of continental East Asia show no significant IBD, which is consistent with previous studies (Gu, Husemann, et al., 2015; Gu, Zhang, et al., 2015; Gu, Zhou, et al., 2015). Our hypothesis that more human use of *S. quadrata* in continental East Asia is likely to contribute to human‐vectored dispersal in modern times is also consistent with the explanation of human transportation (Gu, Husemann, et al., 2015; Gu, Zhang, et al., 2015; Gu, Zhou, et al., 2015). In addition, comparing with previous studies, our comparison study under different situations of human use between continental East Asia and Japan promotes to understand that human‐vectored dispersal could increase the genetic connectivity (Bilton, Freeland, & Okamura, 2001; Holland & Cowie, 2007; Maguire, 1963).

### 4.3 Long‐term geographical isolation in Japan and modern human use in continental east Asia

Populations in a small habitat area generally have a relatively low genetic diversity (Andrén, 1994; Bridle, Pedro, & Butlin, 2004; Tews et al., 2003). The introduced populations (here they are the populations in Japan) might also be expected to be less structured than the source populations (Dlugosch & Parker, 2008; Dybdahl & Drown, 2011; Mergeay et al., 2006; Vogel et al., 2010). However, here, we detected the opposite case, and we generated a hypothesis that populations of *S. quadrata* on relatively small scale in Japan had lower genetic diversity but stronger population structure than that in continental East Asia. Populations in the continental East Asia showed high genetic diversity but weak structure, which is consistent with previous studies (Gu, Husemann, et al., 2015; Gu, Zhang, et al., 2015; Gu, Zhou, et al., 2015). We supplemented the comparison study of populations in Japan and agreed in principle with the effects that are mentioned in previous studies, but we also observed that the uniform genetic structure for contemporary populations in continental East Asia is mainly shaped by modern human use, while that of the higher structure in Japan is mainly shaped by long‐term geographical isolation.

For the human effects, *S. quadrata* is not food or a food product that was eaten by Japanese people. It seems hard to compare the details of food consumption of this snail and to detect the difference in the transportation system between Japan and continental East Asia, but human use and disturbance suggested that human‐vectored dispersal across continental East Asia is higher, resulting in the higher gene flow. For populations in Japan, the genetic structure seems to be correlated with the geological structure of the country. The Japanese archipelago contains four main islands, including Hokkaido (not involved in these studies), Honshu, Shikoku, and Kyushu from east to west. The largest island of Honshu is usually divided into two regions of Kanto (east) and Kansai (west) by mountains between Lake Biwa and Nagoya, and the Kanto region also contains an area of Tohoku (northeast) (Figure 6a). Our results suggest that population divergences in Japan are largely consistent with common geographical boundaries. For example, structure clusters in Japan are divided into regions around Nagoya and Biwako (Figure 1b) where the boundary between the two regions of Kanto (east) and Kansai (west) in Honshu is located (Figure 6a). In addition, population divergences in Japan are significantly correlated with longitude in the direction of the east–west geographical divisions (Figure 4e). Although the demographic history showed that historical gene flow might happen among these regions, the isolated geographical patterns and lack of human disturbance probably hide the effects of gene flow for contemporary populations in Japan. We suggest that the population genetics are more differentiated in Japan because of the combined effects of less human use and more geographical divergence. Therefore, long‐term geographical isolation and modern human use show trends toward becoming the corresponding focal point that affect the genetic patterns of the contemporary populations in Japan and continental East Asia.

In conclusion, *S. quadrata* populations historically had two main approaches to migration from continental East Asia to Japan. One approach is natural migration that is associated with the geohistory of Japan in ancient times, and the other approach is the artificial introduction that is associated with agriculture expansion by human movements in recent times. Populations in Kyushu were initially derived from the continent probably through the East China Sea land bridge during the last glacial period (about 70,000 years ago) as the first approach. Populations in eastern Japan originated recently (about 8,000 years ago) from continental East Asia and Kyushu at the beginning of the agriculture expansion through human movement from continental East Asia to Japan. Populations in western Japan, however, originated more recently (about 1,200 year ago) from continental East Asia and eastern Japan when the socioeconomic and culture was comprehensively exchanged between continental East Asia (such as China) and Japan. Natural migration in the ancient period and artificial introduction in the recent period suggest that the population distribution of *S. quadrata* is affected by both the geohistory of East Asia and the historical human expansion. Moreover, in the background of the historical migration and introduction, contemporary populations in the two regions may experience different biogeographic processes, which are manifested in different genetic structures in the two regions. Populations in Japan that have complex geographical barriers are more highly structured and isolated by distance than that in their source populations in continental East Asia where there is more human use of this snail, suggesting that long‐term geographical isolation is likely the major factor that shapes the genetic structure of contemporary populations in Japan, while modern human uses are likely the major factors affecting the genetic structure in continental East Asia. Although further studies are required, our preliminary results reveal a complex population history and unusual genetic patterns in the contemporary populations for a common freshwater snail in Japan and continental East Asia. By emphasizing the effects of natural processes and humans, our study is of significance to research on the historical formation and contemporary patterns of biogeography in East Asia.

## CONFLICT OF INTERESTS

The authors declare that they have no competing interests.

## AUTHOR CONTRIBUTION

**Bin Ye:** Conceptualization (lead); Data curation (lead); Formal analysis (lead); Resources (equal); Software (lead); Validation (lead); Visualization (lead); Writing‐original draft (lead); Writing‐review & editing (lead). **Takumi Saito:** Conceptualization (equal); Data curation (equal); Investigation (lead); Resources (equal); Software (equal); Writing‐original draft (equal); Writing‐review & editing (equal). **Takahiro Hirano:** Conceptualization (equal); Data curation (equal); Investigation (lead); Resources (equal); Software (equal); Writing‐original draft (equal); Writing‐review & editing (equal). **Zhengzhong Dong:** Investigation (equal); Resources (equal); Writing‐original draft (equal); Writing‐review & editing (equal). **Van Tu Do:** Investigation (equal); Resources (equal); Writing‐original draft (equal); Writing‐review & editing (equal). **Satoshi Chiba:** Conceptualization (lead); Data curation (equal); Formal analysis (equal); Funding acquisition (lead); Project administration (lead); Resources (equal); Writing‐original draft (equal); Writing‐review & editing (equal).

## Supporting information

Supinfo S1

Supinfo S2

Supinfo S3

## Data Availability

Appendix S1, Table files for Table S1–S9; Appendix S2, Figure files for Figure S1–S8; Appendix S3, population *F_IS_* and environmental variables; Appendix S4, Genotype file for analysis in DIYABC; and data of microsatellite genotypes are deposited in Dryad: http://doi.org/10/5061/dryad.h18931zgk
