# Supplementary material for: Human‐geographic effects on variations in the population genetics of Sinotaia quadrata (Gastropoda: Viviparidae) that historically migrated from continental East Asia to Japan
Source: Ecol Evol. 2020 Jul 15;10(15):8055–72. doi: 10.1002/ece3.6456 (PMC7417235; doi:10.1002/ece3.6456)
Supplement: Supplementary file 1 — Supinfo S1 [file ECE3-10-8055-s001.pdf]

Appendix S1

for

Human-geographic effects on variations in the population genetics of *Sinotaia quadrata* (Gastropoda: Viviparidae) that were historically introduced from the continental East Asia to Japan

Bin Ye, Takumi Saito, Takahiro Hirano, Zhengzhong Dong, Van Tu Do, Satashi Chiba

Table S1 Twelve microsatellite loci primers used for *Sinotaia quadrata*.

Table S2 Null allele frequency for each locus and population.

Table S3 Variables used for analysis of the relationship between human disturbance of the environment and population divergence.

Table S4 Significant variables correlating with allele frequency in RDA (999 permutations).

Table S5 Historical parameters priors set for scenarios in DIYABC.

Table S6 *P*-values of linkage disequilibrium (LD) between pairwise loci in total dataset.

Table S7 Comparison of scenarios using logistic approach estimated by DIYABC.

Table S8 Historical parameters posteriors for scenario 11 and scenario 7 estimated by DIYABC.

Table S9 Scenario specific prior based error for scenario 11 estimated using logistic approach. Type I error, the probability with which it is rejected although it is the true scenario. Type II error, probability of deciding for the target scenario when it is not the true scenario.

Table S1 Twelve microsatellite loci primers used for *Sinotaia quadrata*

Locus	Flurescent label	Primer (5' to 3')*		GenBank
ATG142	FAM	F:	ATGCCGATTATTCTGATTCTGG	JX019326
	-	R:	GTTTCTTACGCAAGTTTCATTCATGTATGTC	
TC298	VIC	F:	CTCCAAAGACTGTTACTGCTACGA	JX019072
	-	R:	GTTTCTTCACACAAACTAGGTAAGGGGACAT	
TXH79	NED	F:	TCCTGATTCAAGGACGTTGTGC	JN555804
	-	R:	GTTTCTTGACTGGTGCTGGCTGTAGTGC	
TXH12	PET	F:	AGGCCTCAGCTTGAATCCCTA	JN555765
	-	R:	GTTTCTTCGGCTCCCATTTTGAGCATTG	
GATA328	FAM	F:	CCTGCGTCAATTTAAAACCATAG	JX020456
	-	R:	GTTTCTTGGGTAGGTAGGTGGGTAAGTGAG	
TXH223	VIC	F:	GCTTGCCACCGCATTGTAGC	JN555901
	-	R:	GTTTCTTCACAACTTGGTCACAAAGCCG	
CAG41	NED	F:	TTTGCTGCGTTTACTCGTCCTG	JX019413
	-	R:	GTTTCTTCTCGTTCTTGGGCTGGGTGTT	
TXH30	PET	F:	CACATAGAAGGTCACACGT	JN555777
	-	R:	GTTTCTTGAATTCCAAACTCAGACAACGG	
CAG178	FAM	F:	CATGACATAACACCCCTACCCTT	JX020456
	-	R:	GTTTCTTAATTTGGTATCTTGGAATCTGACG	
TXH65	VIC	F:	TGTGACTAAGTGTGTTTGCATG	JN580068
	-	R:	GTTTCTTCTACCAGGTGCTTGGTGTG	
CCT238	NED	F:	ACATAAGTGCTTGCGATAGTGCG	JX019326
	-	R:	GTTTCTTCTACCAGGTGCTTGGTGTG	
TXH113	PET	F:	CAAGCATGGATGCAGAACTC	JN555824
	-	R:	GTTTCTTCTCGTTGGTCCGATACAACC	

* PIG-tail 5’-GTTTCTT-3’ is added at the 5’ end of each reserve primer.

Table S2 Null allele frequency for each locus and population

Site	ATG142	TC298	TXH79	TXH12	GATA328	TXH223	CAG41	TXH30	CAG178	TXH65	CCT238	TXH113
AM	0.07	0.02	0.16	0.22	0.00	0.11	0.08	0.13	0.31	0.08	0.03	0.00
AK	0.05	0.06	0.10	0.00	0.06	0.12	0.26	0.35	0.10	0.12	0.00	0.08
MY	0.00	0.12	0.00	0.00	0.00	0.15	0.06	0.29	0.00	0.01	0.22	0.01
FK	0.05	0.06	0.26	0.17	0.10	0.18	0.26	0.10	0.17	0.06	0.03	0.18
NGN	0.00	0.00	0.20	0.02	0.07	0.00	0.02	0.12	0.15	0.01	0.00	0.00
KN	0.10	0.16	0.31	0.04	0.00	0.22	0.08	0.24	0.05	0.00	0.16	0.03
SZ	0.00	0.15	0.12	0.03	0.00	0.04	0.00	0.12	0.15	0.00	0.00	0.15
NGY	0.00	0.04	0.18	0.02	0.04	0.11	0.16	0.17	0.11	0.03	0.14	0.01
SG	0.01	0.00	0.12	0.08	0.00	0.20	0.00	0.06	0.22	0.00	0.00	0.05
BW	0.00	0.00	0.27	0.07	0.00	0.12	0.14	0.04	0.14	0.01	0.00	0.02
NR	0.00	0.00	0.00	0.00	0.00	0.24	0.06	0.00	0.21	0.01	0.01	0.03
OS	0.00	0.16	0.27	0.04	0.16	0.12	0.04	0.04	0.06	0.14	0.10	0.05
KGW	0.05	0.09	0.14	0.09	0.09	0.09	0.10	0.29	0.17	0.07	0.13	0.14
KC	0.00	0.00	0.11	0.05	0.07	0.12	0.05	0.09	0.00	0.03	0.05	0.05
YM	0.16	0.05	0.19	0.00	0.11	0.01	0.16	0.14	0.27	0.10	0.20	0.28
KT	0.00	0.04	0.16	0.11	0.00	0.05	0.13	0.14	0.05	0.11	0.14	0.18
NGS	0.00	0.00	0.13	0.06	0.00	0.14	0.06	0.29	0.09	0.16	0.24	0.27
KGS	0.32	0.00	0.00	0.00	0.12	0.05	0.00	0.00	0.17	0.08	0.11	0.18
CC	0.14	0.27	0.13	0.06	0.18	0.01	0.11	0.24	0.09	0.12	0.17	0.15
AN	0.00	0.07	0.14	0.14	0.15	0.00	0.15	0.14	0.05	0.16	0.28	0.11
NJ	0.04	0.05	0.20	0.17	0.13	0.10	0.03	0.17	0.18	0.14	0.19	0.02
HZ	0.02	0.13	0.16	0.11	0.12	0.10	0.19	0.12	0.21	0.12	0.20	0.06
CA	0.08	0.00	0.19	0.11	0.20	0.00	0.09	0.00	0.05	0.00	0.30	0.04
HB	0.09	0.03	0.21	0.22	0.26	0.11	0.14	0.17	0.31	0.16	0.11	0.06
JJ	0.05	0.07	0.20	0.06	0.18	0.00	0.00	0.28	0.15	0.15	0.16	0.05
SC	0.27	0.24	0.15	0.00	0.13	0.25	0.20	0.26	0.15	0.12	0.11	0.06
YZH	0.10	0.03	0.09	0.13	0.16	0.12	0.16	0.01	0.02	0.10	0.00	0.08
SL	0.00	0.16	0.04	0.10	0.14	0.00	0.00	0.07	0.07	0.01	0.06	0.04
HK	0.12	0.05	0.15	0.12	0.08	0.15	0.07	0.24	0.13	0.19	0.21	0.09
YL	0.00	0.00	0.08	0.04	0.05	0.08	0.18	0.03	0.13	0.03	0.14	0.00
TN	0.11	0.00	0.10	0.14	0.00	0.00	0.08	0.14	0.00	0.14	0.00	0.07
CB	0.00	0.00	0.25	0.00	0.13	0.00	0.00	0.00	0.00	0.00	0.00	0.21
LS	0.14	0.08	0.23	0.15	0.22	0.01	0.22	0.07	0.31	0.12	0.00	0.16

Table S3 Variables used for analysis of the relationship between human disturbance of the environment and population divergence.

Variables	Explanation	Variables	Explanation
PopD1km	Human population density with buffer radii of 1 km around each sampling location	BIO1	Annual Mean Temperature
PopD5km	Human population density with buffer radii of 5 km around each sampling location	BIO2	Mean Diurnal Range (Mean of monthly (max temp - min temp))
PopD10km	Human population density with buffer radii of 10 km around each sampling location	BIO3	Isothermality (BIO2/BIO7) (×100)
PopD20km	Human population density with buffer radii of 20 km around each sampling location	BIO4	Temperature Seasonality (standard deviation ×100)
PopD50km	Human population density with buffer radii of 50 km around each sampling location	BIO5	Max Temperature of Warmest Month
HmFp1km	Human footprint pressure with buffer radii of 1 km around each sampling location	BIO6	Min Temperature of Coldest Month
HmFp5km	Human footprint pressure with buffer radii of 5 km around each sampling location	BIO7	Temperature Annual Range (BIO5-BIO6)
HmFp10km	Human footprint pressure with buffer radii of 10 km around each sampling location	BIO8	Mean Temperature of Wettest Quarter
HmFp20km	Human footprint pressure with buffer radii of 20 km around each sampling location	BIO9	Mean Temperature of Driest Quarter
HmFp50km	Human footprint pressure with buffer radii of 50 km around each sampling location	BIO10	Mean Temperature of Warmest Quarter
UbEx20km	Urban expansion percentage with buffer radii of 20 km around each sampling location	BIO11	Mean Temperature of Coldest Quarter
UbEx50km	Urban expansion percentage with buffer radii of 20 km around each sampling location	BIO12	Annual Precipitation
NetM20km	Human net migration with buffer radii of 20 km around each sampling location	BIO13	Precipitation of Wettest Month
NetM50km	Human net migration with buffer radii of 50 km around each sampling location	BIO14	Precipitation of Driest Month
Longitude	Longitude of each sampling location	BIO15	Precipitation Seasonality (Coefficient of Variation)
Latitude	Latitude of sampling location	BIO16	Precipitation of Wettest Quarter
		BIO17	Precipitation of Driest Quarter
		BIO18	Precipitation of Warmest Quarter
		BIO19	Precipitation of Coldest Quarter

Table S4 Significant variables correlating with allele frequency in RDA (999 permutations).

Group	Significant variables	*df*	*variance*	*F*	*p*
Total	HmFp1km + UbEx20km + UbEx50km + BIO4 + BIO10 + BIO11 + BIO18 + Longitude	8	0.7661	1.6558	0.001 ***
Japan	UbEx20km + UbEx50km + BIO4 + BIO11 + Longitude	5	1.0720	1.7405	0.001 ***
China-Vietnam	UbEx50km + BIO3 + BIO4 + BIO5 + BIO7 + BIO11 + BIO14 + BIO19 + Latitude	9	1.0702	1.8503	0.004 **

*** $p < 0.001$; ** $p < 0.01$

Table S5 Historical parameters priors set for scenarios in DIYABC

Parameters*		Distribution	Minimum	Maximum
N1	Population size of JP1 at T1	Uniform	10.0	200000
N2	Population size of JP2 at T1	Uniform	10.0	200000
N3	Population size of JPK at T1	Uniform	10.0	200000
N4	Population size of CN at T1	Uniform	10.0	800000
N1.1	Population size of JP1 at T2	Uniform	10.0	100000
N2.1	Population size of JP2 at T2	Uniform	10.0	100000
N3.1	Population size of JPK at T2	Uniform	10.0	100000
N4.1	Population size of CN at T2	Uniform	10.0	200000
N3.2	Population size of JPK at T3	Uniform	10.0	20000
N4.2	Population size of CN at T3	Uniform	10.0	50000
NA	Population size of ancestor	Uniform	10.0	10000
T1	Effective number of generations (newest)	Uniform	10.0	10000
T1.1	Effective number of generations	Uniform	10.0	20000
T2	Effective number of generations	Uniform	10.0	50000
T2.1	Effective number of generations	Uniform	10.0	100000
T3	Effective number of generations (oldest)	Uniform	0.001	200000
r14	Admixture between JP1 and CN	Uniform	0.001	0.999
r24	Admixture between JP2 and CN	Uniform	0.001	0.999
r34	Admixture between JPK and CN	Uniform	0.001	0.999
r13	Admixture between JP1 and JPK	Uniform	0.001	0.999
r23	Admixture between JP2 and JPK	Uniform	0.001	0.999

* With conditions: T3 >= T2.1 > T2 >= T1.1 > T1, N1 >= N1.1, N2 >= N2.1, N4 >= N4.1 >= N4.2 > NA, N3 >= N3.1 >= N3.2 > NA

Table S6 *P*-values of linkage disequilibrium (LD) between pairwise loci in total dataset.

	ATG142	TC298	TXH79	TXH12	GATA328	TXH223	CAG41	TXH30	CAG178	TXH65	CCT238
TC298	0.38										
TXH79	0.00*	0.55									
TXH12	0.00*	0.10	0.00*								
GATA328	1.00	0.99	0.37	0.89							
TXH223	0.93	0.78	0.22	0.94	0.05*						
CAG41	0.37	0.15	0.98	0.51	0.11	0.00*					
TXH30	0.73	1.00	0.72	0.89	0.69	0.09	0.98				
CAG178	0.97	0.97	0.30	0.34	0.36	0.41	0.27	0.63			
TXH65	0.55	0.65	0.16	0.93	0.02*	0.09	0.23	0.41	0.02*		
CCT238	0.70	0.90	0.55	0.45	0.99	0.94	0.84	0.97	0.00*	0.00*	
TXH113	1.00	1.00	0.84	0.91	0.46	0.75	0.99	0.99	0.79	0.99	0.23

* $p < 0.05$

Table S7 Comparison of scenarios using logistic approach estimated by DIYABC

n	scenario 1	scenario 2	scenario 3	scenario 4
20000	0.0049 [0.0000,0.0343]	0.0184 [0.0000,0.0471]	0.0063 [0.0000,0.0356]	0.0154 [0.0000,0.0442]
40000	0.0052 [0.0000,0.0271]	0.0191 [0.0000,0.0403]	0.0065 [0.0000,0.0283]	0.0155 [0.0000,0.0369]
60000	0.0051 [0.0000,0.0234]	0.0185 [0.0007,0.0362]	0.0064 [0.0000,0.0246]	0.0155 [0.0000,0.0333]
80000	0.0051 [0.0000,0.0210]	0.0181 [0.0026,0.0336]	0.0062 [0.0000,0.0221]	0.0155 [0.0000,0.0311]
100000	0.0049 [0.0000,0.0193]	0.0177 [0.0037,0.0317]	0.0059 [0.0000,0.0203]	0.0154 [0.0014,0.0295]
120000	0.0048 [0.0000,0.0181]	0.0173 [0.0045,0.0302]	0.0057 [0.0000,0.0190]	0.0153 [0.0024,0.0282]
140000	0.0047 [0.0000,0.0171]	0.0171 [0.0050,0.0291]	0.0056 [0.0000,0.0180]	0.0151 [0.0030,0.0272]
160000	0.0046 [0.0000,0.0164]	0.0169 [0.0055,0.0282]	0.0055 [0.0000,0.0172]	0.0149 [0.0035,0.0263]
180000	0.0046 [0.0000,0.0157]	0.0166 [0.0058,0.0275]	0.0053 [0.0000,0.0165]	0.0147 [0.0039,0.0256]
200000	0.0045 [0.0000,0.0152]	0.0164 [0.0061,0.0268]	0.0052 [0.0000,0.0158]	0.0146 [0.0041,0.0250]
n	scenario 5	scenario 6	scenario 7	scenario 8
20000	0.0211 [0.0000,0.0496]	0.1299 [0.0711,0.1887]	**0.3361 [0.2823,0.3900]**	0.0002 [0.0000,0.0300]
40000	0.0201 [0.0000,0.0413]	0.1150 [0.0796,0.1505]	**0.3275 [0.2900,0.3649]**	0.0002 [0.0000,0.0225]
60000	0.0193 [0.0016,0.0370]	0.1175 [0.0894,0.1456]	**0.3245 [0.2938,0.3552]**	0.0003 [0.0000,0.0189]
80000	0.0184 [0.0029,0.0339]	0.1177 [0.0941,0.1413]	**0.3261 [0.2996,0.3526]**	0.0003 [0.0000,0.0165]
100000	0.0174 [0.0034,0.0314]	0.1213 [0.1003,0.1422]	**0.3254 [0.3018,0.3491]**	0.0003 [0.0000,0.0150]
120000	0.0164 [0.0036,0.0293]	0.1254 [0.1062,0.1445]	**0.3248 [0.3032,0.3464]**	0.0003 [0.0000,0.0138]
140000	0.0155 [0.0035,0.0276]	0.1287 [0.1110,0.1465]	**0.3237 [0.3036,0.3438]**	0.0003 [0.0000,0.0129]
160000	0.0148 [0.0034,0.0262]	0.1323 [0.1156,0.1490]	**0.3218 [0.3029,0.3406]**	0.0003 [0.0000,0.0122]
180000	0.0142 [0.0033,0.0250]	0.1358 [0.1200,0.1517]	**0.3203 [0.3025,0.3381]**	0.0003 [0.0000,0.0116]
200000	0.0136 [0.0032,0.0240]	0.1385 [0.1234,0.1536]	**0.3191 [0.3021,0.3360]**	0.0003 [0.0000,0.0111]
n	scenario 9	scenario 10	scenario 11	scenario 12
20000	0.0122 [0.0000,0.0410]	0.1318 [0.0716,0.1920]	**0.3236 [0.2786,0.3686]**	0.0002 [0.0000,0.0301]
40000	0.0139 [0.0000,0.0354]	0.1434 [0.1012,0.1856]	**0.3334 [0.3002,0.3666]**	0.0004 [0.0000,0.0226]
60000	0.0148 [0.0000,0.0327]	0.1407 [0.1084,0.1729]	**0.3372 [0.3095,0.3648]**	0.0004 [0.0000,0.0190]
80000	0.0150 [0.0000,0.0306]	0.1391 [0.1123,0.1659]	**0.3380 [0.3137,0.3623]**	0.0004 [0.0000,0.0166]
100000	0.0145 [0.0005,0.0286]	0.1403 [0.1169,0.1637]	**0.3363 [0.3144,0.3582]**	0.0004 [0.0000,0.0151]
120000	0.0140 [0.0011,0.0269]	0.1418 [0.1207,0.1629]	**0.3337 [0.3136,0.3538]**	0.0004 [0.0000,0.0139]
140000	0.0134 [0.0013,0.0255]	0.1429 [0.1236,0.1622]	**0.3325 [0.3138,0.3512]**	0.0004 [0.0000,0.0130]
160000	0.0129 [0.0015,0.0244]	0.1442 [0.1263,0.1622]	**0.3314 [0.3138,0.3490]**	0.0004 [0.0000,0.0123]
180000	0.0125 [0.0016,0.0234]	0.1455 [0.1287,0.1623]	**0.3298 [0.3131,0.3465]**	0.0004 [0.0000,0.0117]
200000	0.0121 [0.0016,0.0225]	0.1470 [0.1312,0.1629]	**0.3284 [0.3125,0.3443]**	0.0003 [0.0000,0.0112]

Table S8 Historical parameters posteriors for scenario 11 and scenario 7 estimated by DIYABC

	Parameter	Mean	Median	Mode	95% CI
Scenario 11	N1	4.76e+04	**3.24e+04**	1.48e+04	[7.79e+03, 1.67e+05]
	N2	4.58e+04	**3.31e+04**	1.90e+04	[5.41e+03, 1.67e+05]
	N3	1.63e+04	**1.06e+04**	7.62e+03	[3.19e+03, 7.84e+04]
	N4	6.19e+05	**6.42e+05**	7.92e+05	[3.29e+05, 7.94e+05]
	N1.1	2.42e+04	**1.88e+04**	9.59e+03	[2.33e+03, 7.81e+04]
	N4.1	1.55e+05	**1.61e+05**	1.90e+05	[7.91e+04, 1.98e+05]
	N3.2	1.40e+04	**1.55e+04**	1.99e+04	[1.67e+03, 1.99e+04]
	N4.2	3.34e+04	**3.62e+04**	4.71e+04	[4.43e+03, 4.93e+04]
	T1	1.89e+03	**1.16e+03**	2.93e+02	[7.45e+01, 7.81e+03]
	T1.1	7.78e+03	**6.69e+03**	4.29e+03	[1.21e+03, 1.86e+04]
	T2	1.11e+04	**7.91e+03**	4.23e+03	[1.84e+03, 3.87e+04]
	T2.1	5.94e+04	**5.85e+04**	5.66e+04	[2.27e+04, 9.68e+04]
	T3	7.87e+04	**6.93e+04**	5.29e+04	[2.11e+04, 1.81e+05]
	r14	4.02e-01	**3.93e-01**	3.87e-01	[9.44e-02, 7.76e-01]
	r34	5.39e-01	**5.45e-01**	5.62e-01	[1.15e-01, 9.24e-01]
Scenario 7	N1	4.20e+04	**2.70e+04**	1.48e+04	[6.47e+03, 1.62e+05]
	N2	6.34e+04	**5.17e+04**	3.41e+04	[1.01e+04, 1.76e+05]
	N3	1.88e+04	**1.27e+04**	9.33e+03	[3.96e+03, 8.63e+04]
	N4	6.19e+05	**6.44e+05**	7.84e+05	[3.26e+05, 7.94e+05]
	N1.1	2.38e+04	**1.87e+04**	1.16e+04	[2.20e+03, 7.74e+04]
	N4.1	1.65e+05	**1.71e+05**	1.98e+05	[9.89e+04, 1.99e+05]
	N3.2	1.11e+04	**1.16e+04**	1.91e+04	[7.38e+02, 1.96e+04]
	N4.2	3.20e+04	**3.45e+04**	4.36e+04	[3.67e+03, 4.93e+04]
	NA	5.52e+03	**5.75e+03**	9.61e+03	[3.49e+02, 9.81e+03]
	T1	1.76e+03	**1.07e+03**	2.66e+02	[6.30e+01, 7.62e+03]
	T1.1	6.88e+03	**5.65e+03**	3.22e+03	[8.98e+02, 1.82e+04]
	T2	1.25e+04	**9.39e+03**	5.53e+03	[2.19e+03, 4.08e+04]
	T2.1	6.79e+04	**6.87e+04**	6.53e+04	[3.11e+04, 9.81e+04]
	T3	1.01e+05	**9.54e+04**	7.77e+04	[3.51e+04, 1.88e+05]
	r14	3.74e-01	**3.61e-01**	3.65e-01	[8.43e-02, 7.68e-01]
	r34	4.94e-01	**4.92e-01**	4.53e-01	[7.79e-02, 9.21e-01]

Table S9 Scenario specific prior based error for scenario 11 estimated using logistic approach. Type I error, the probability with which it is rejected although it is the true scenario. Type II error, probability of deciding for the target scenario when it is not the true scenario.

Scenario	Type I Error for scenario 11	Type II Error for scenario 11
1	0.022	0.045
2	0.097	0.166
3	0.019	0.024
4	0.115	0.178
5	0.045	0.042
6	0.053	0.054
7	0.449	0.484
8	0.038	0.041
9	0.038	0.037
10	0.061	0.043
12	0.028	0.031
