# Supplementary material for: Human‐geographic effects on variations in the population genetics of Sinotaia quadrata (Gastropoda: Viviparidae) that historically migrated from continental East Asia to Japan
Source: Ecol Evol. 2020 Jul 15;10(15):8055–72. doi: 10.1002/ece3.6456 (PMC7417235; doi:10.1002/ece3.6456)
Supplement: Supplementary file 2 — Supinfo S2 [file ECE3-10-8055-s002.pdf]

Appendix S2

for

Human-geographic effects on variations in the population genetics of *Sinotaia quadrata* (Gastropoda: Viviparidae) that were historically introduced from the continental East Asia to Japan

Bin Ye, Takumi Saito, Takahiro Hirano, Zhengzhong Dong, Van Tu Do, Satashi Chiba

Figure S1 Data extraction using buffer radiuses limited up to 50 km (the solid circle) around each location. Buffer radiuses larger than 50 km (e.g. 100 km, the dot circle) would cause more overlaps of data for closed localities.

Figure S2 Optimal α score of retained PCs for DAPC with prior information.

Figure S3 DAPC of total, Japan, and China-Vietnam with no priori information. Each row from left to right: Plot of Bayesian information criterion (BIC) scores for K from 1 to 30, inferred clusters identified from actual populations, DAPC plot with no priori information, discriminant density plot. Each column from up to down: total, Japan, China-Vietnam.

Figure S4 *DeltaK* values from Structure analyses.

Figure S5 Posterior probabilities of scenarios computed using linear discriminant analysis on summary statistics.

Figure S6 Scaled posterior distribution of parameters estimated by logit transformation for scenario 11

Figure S7 Scaled posterior distribution of parameters estimated by logit transformation for scenario 7

Figure S8 PCA form scenario 11 on model checking

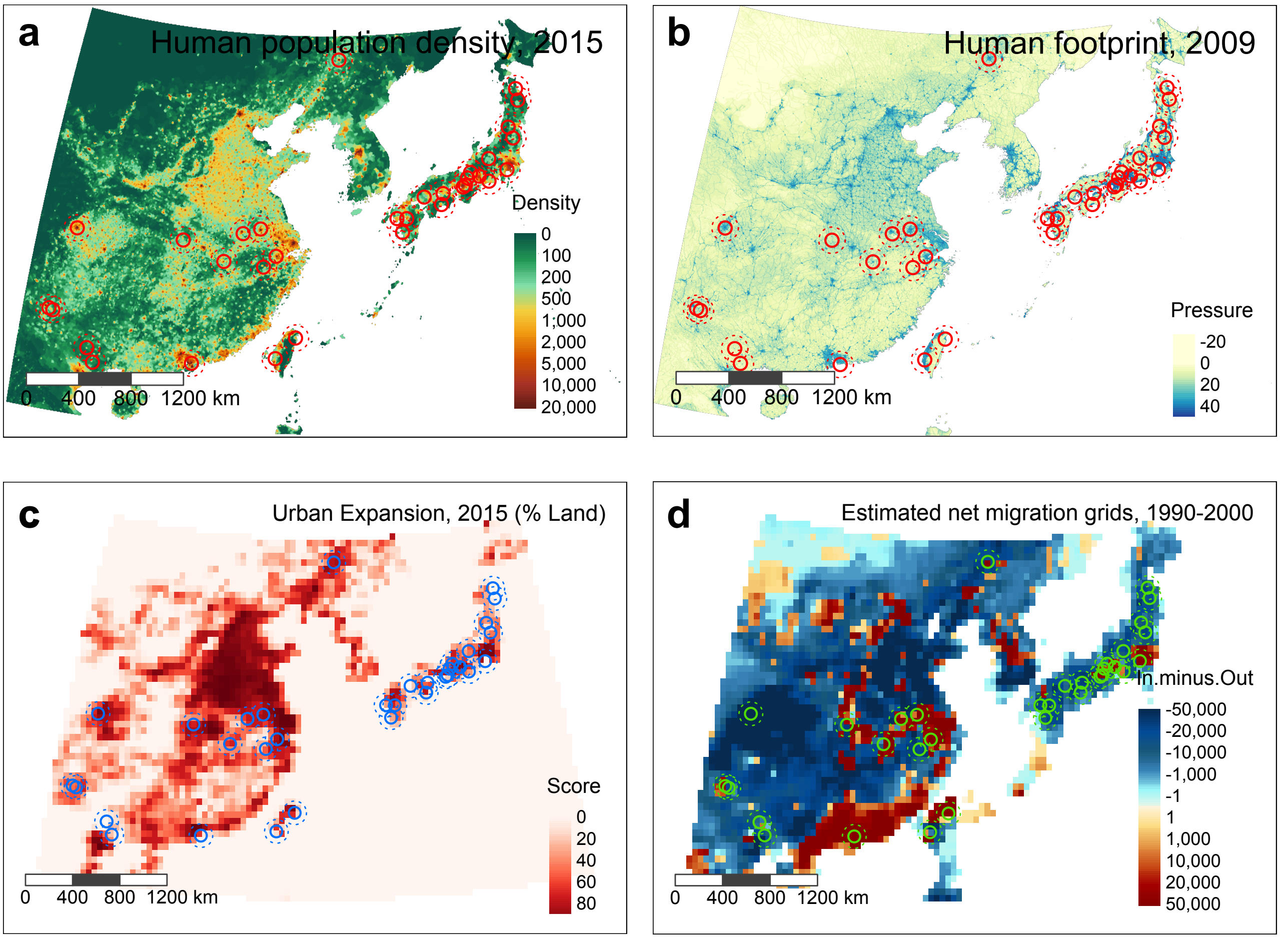

Figure S1 Data extraction using buffer radiuses limited up to 50 km (the solid circle) around each locality. Buffer radiuses larger than 50 km (e.g. 100 km, the dot circle) would cause more overlaps of data for closed localities.

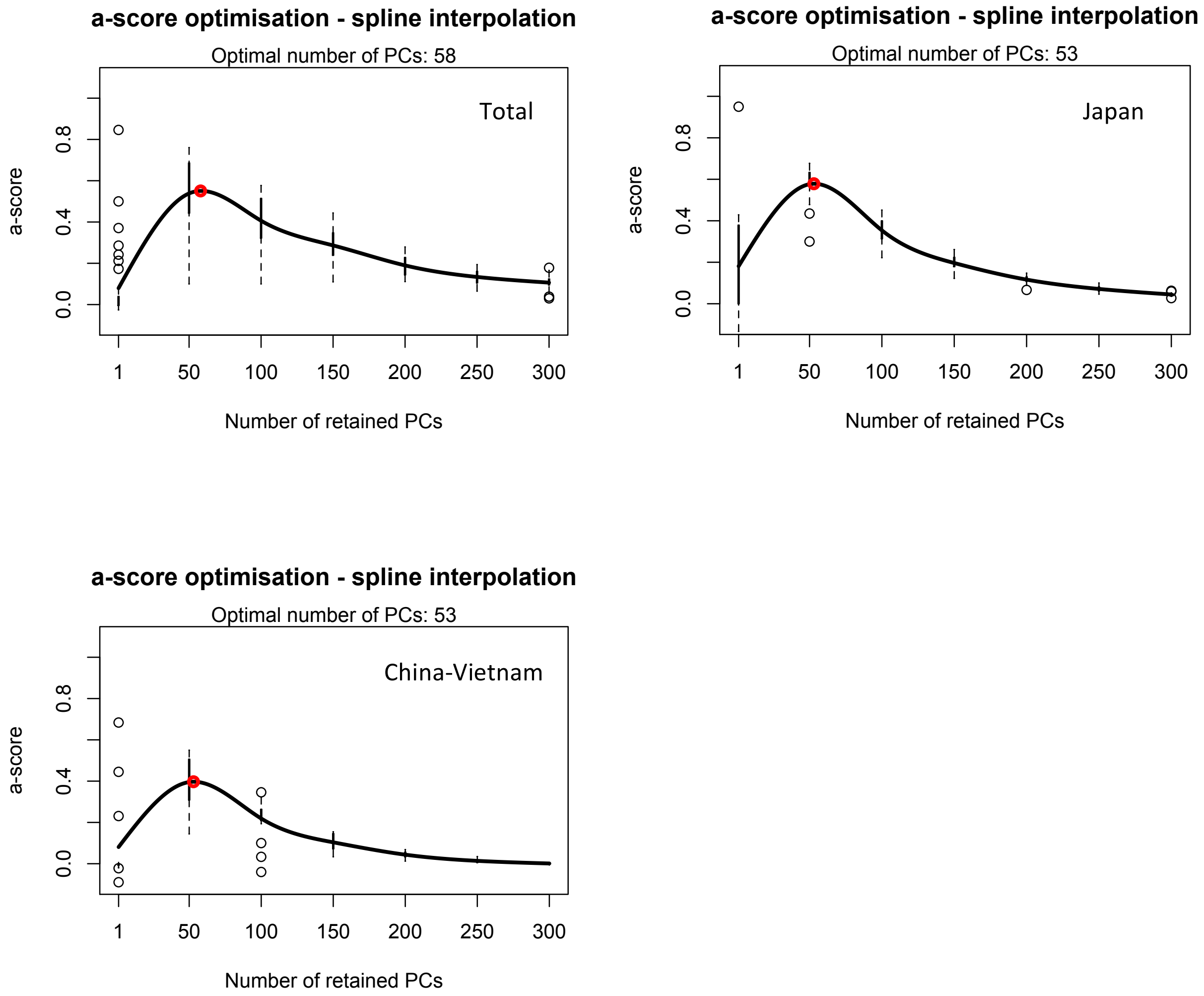

Figure S2 Optimal α score of retained PCs for DAPC with prior information.

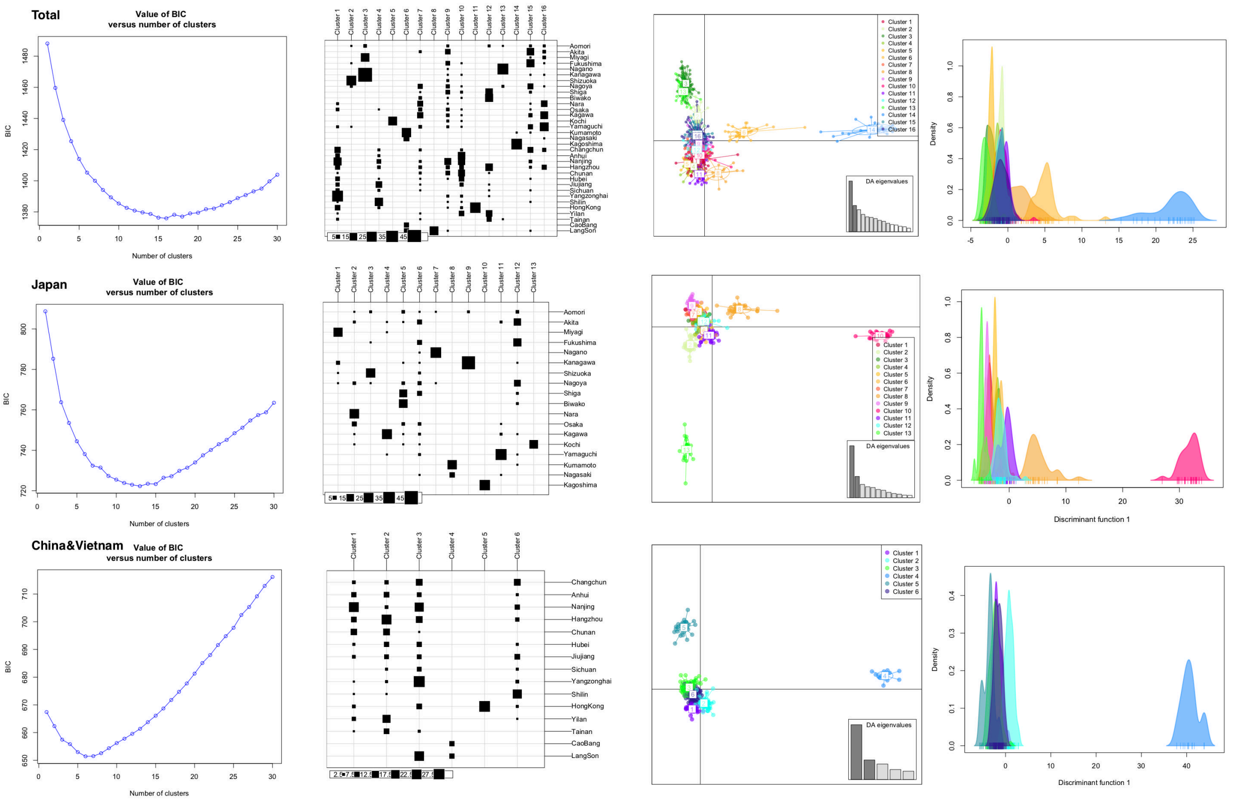

Figure S3 DAPC of total, Japan, and China&Vietnam with no priori information. Each row from left to right: Plot of Bayesian information criterion (BIC) scores for K from 1 to 30, inferred clusters identified from actual populations, DAPC plot with no priori information, discriminant density plot. Each column from up to down: total, Japan, China&Vietnam.

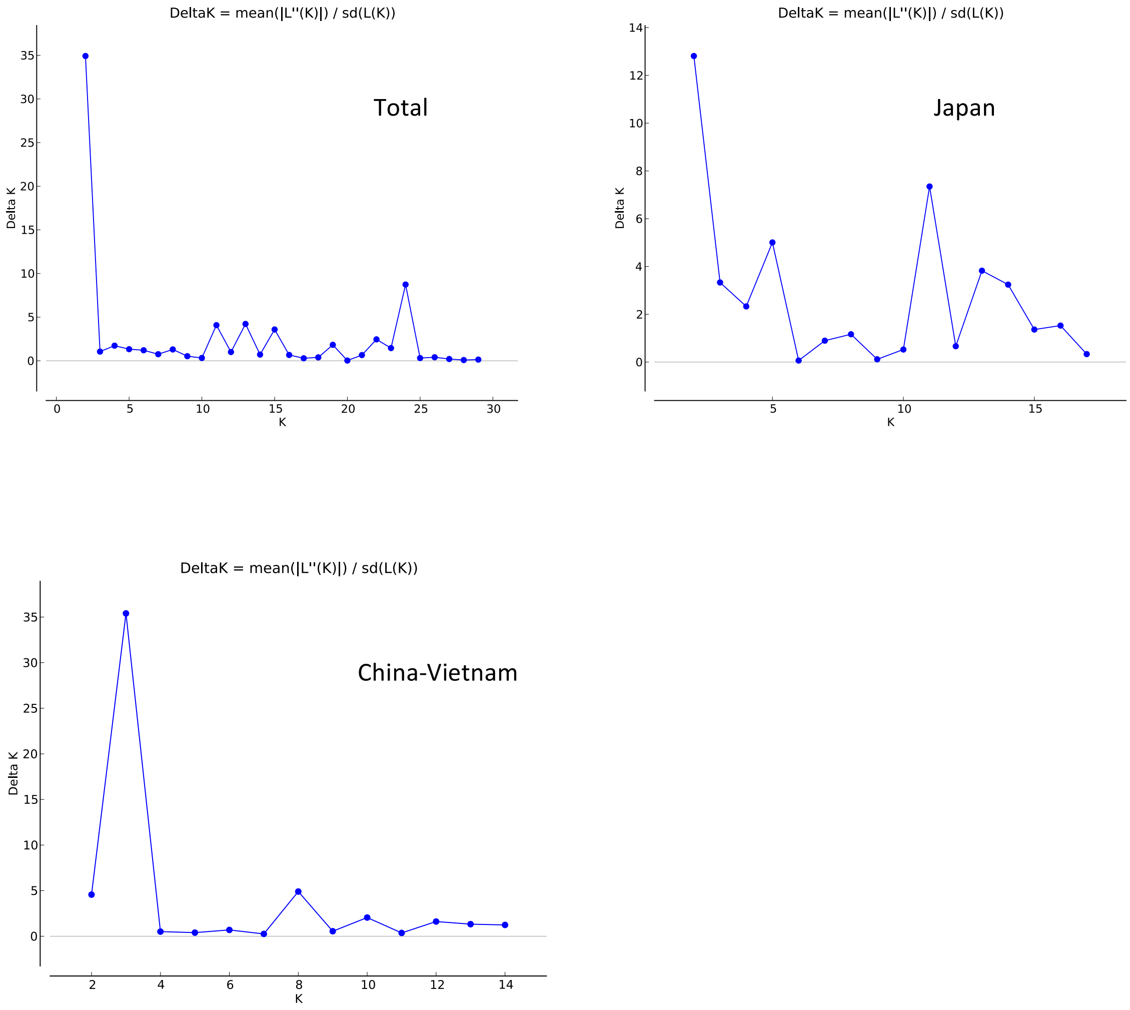

Figure S4 *DeltaK* values from Structure analyses.

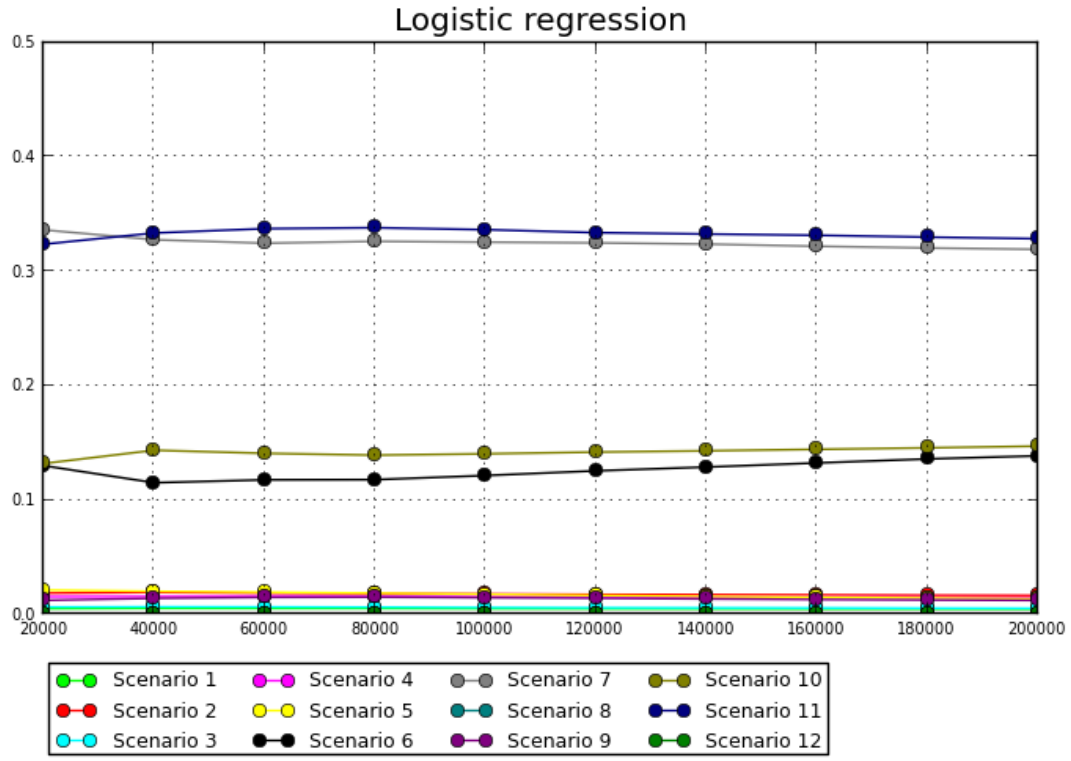

Figure S5 Posterior probabilities of scenarios computed using linear discriminant analysis on summary statistics.

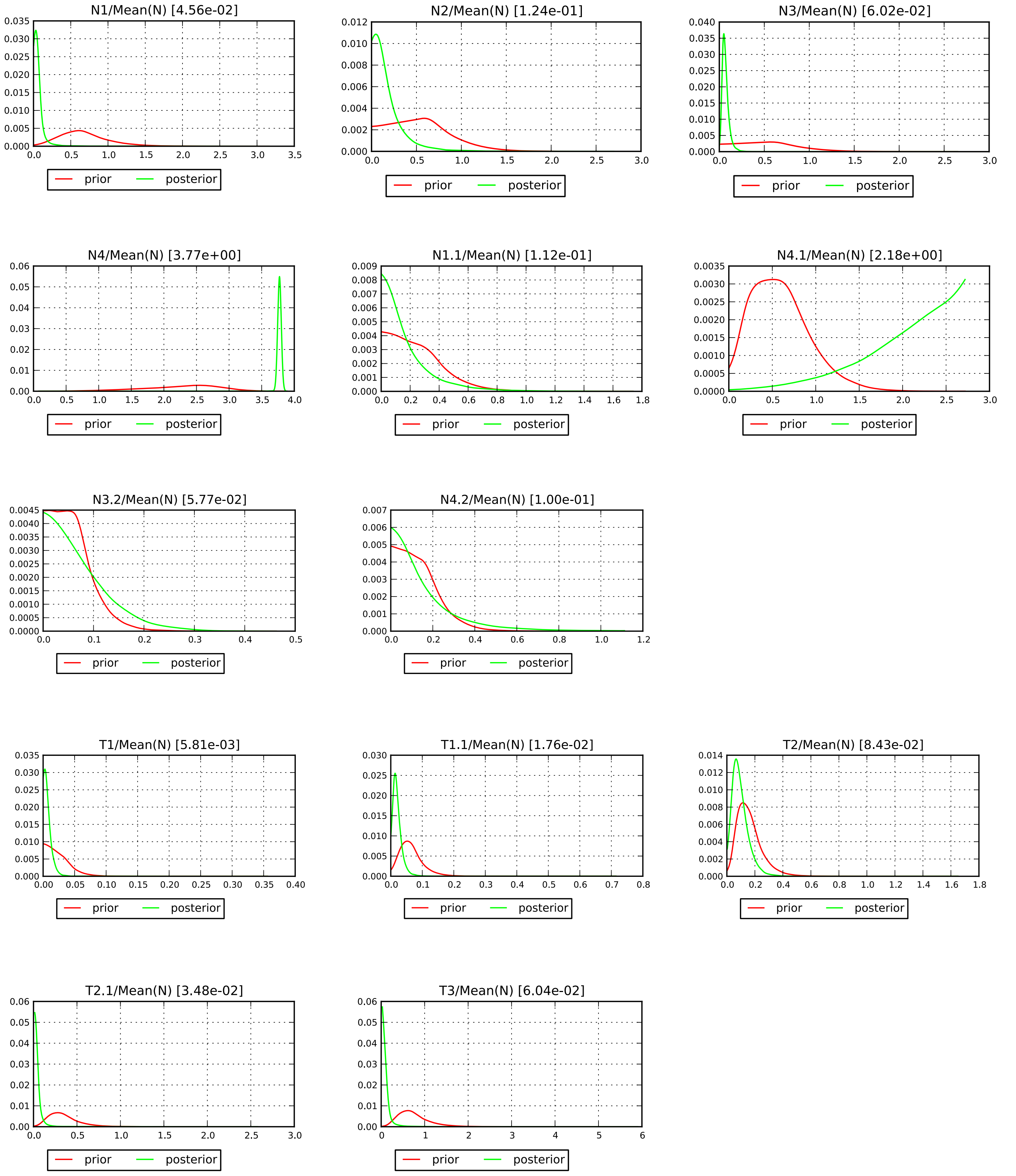

Figure S6 Scaled posterior distribution of parameters estimated by logit transformation for scenario 11

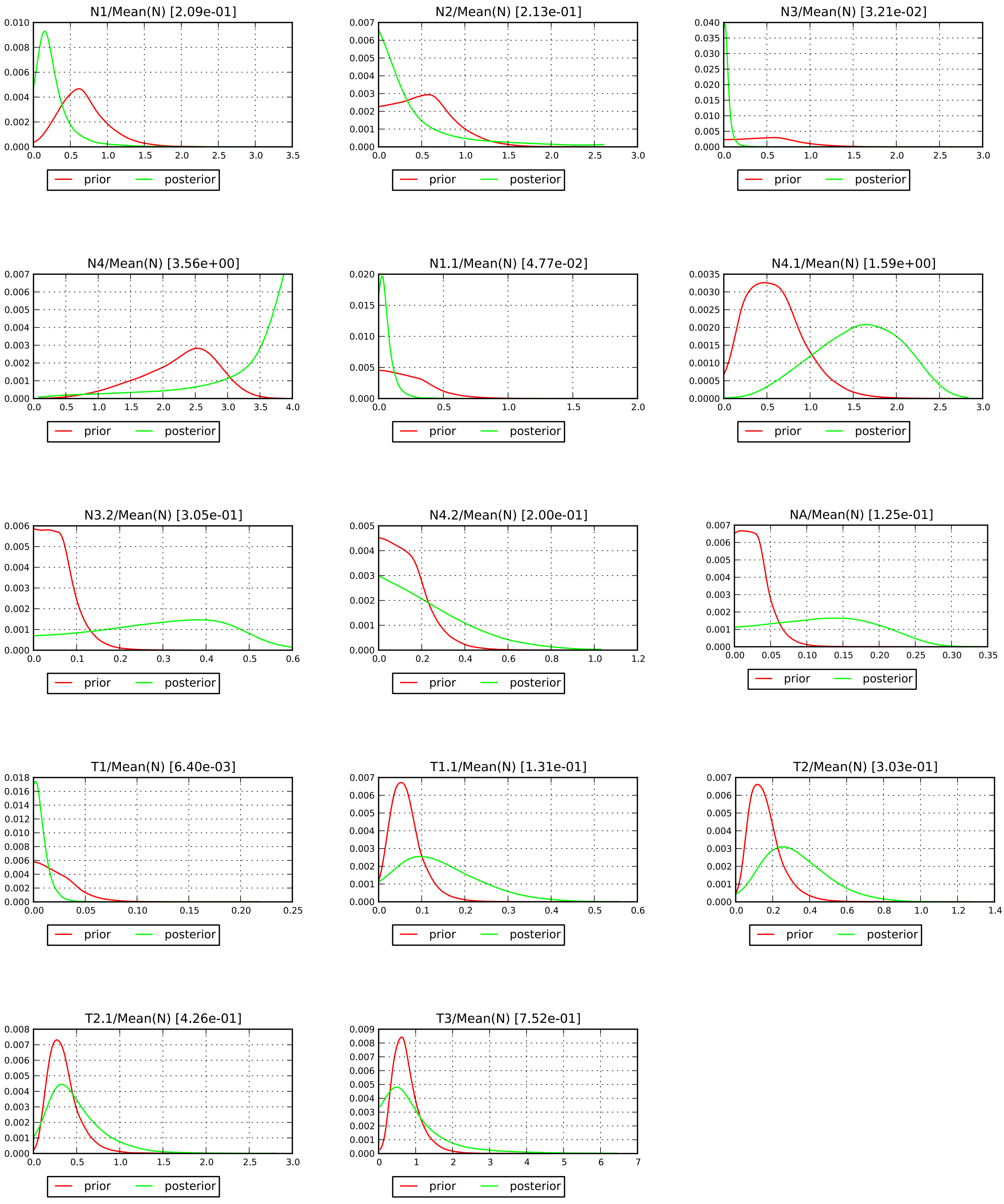

Figure S7 Scaled posterior distribution of parameters estimated by logit transformation for scenario 7

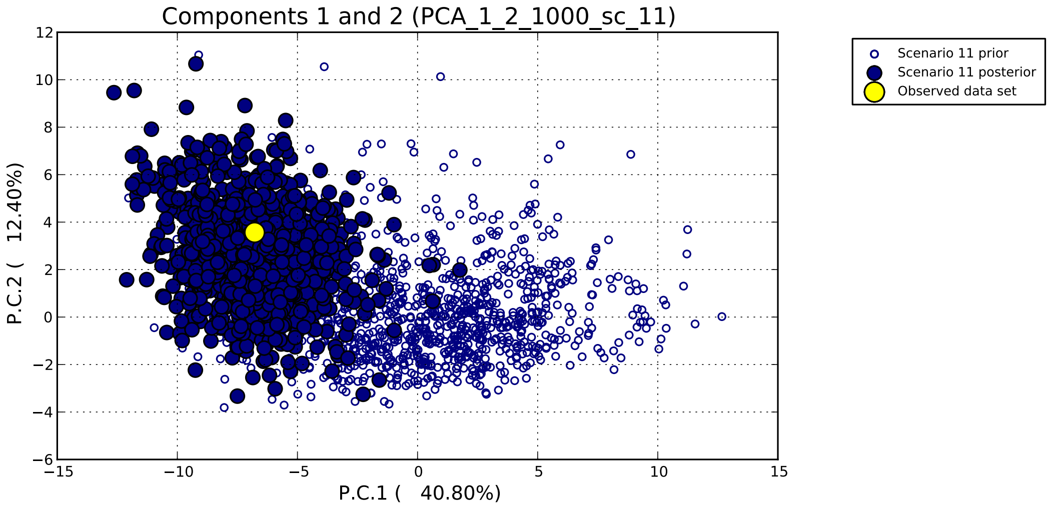

Figure S8 PCA form scenario 11 on model checking
